# CPR-C4 is a highly conserved novel protease from the Candidate Phyla Radiation with remote structural homology to human vasohibins

**DOI:** 10.1016/j.jbc.2022.101919

**Published:** 2022-04-08

**Authors:** Katy A.S. Cornish, Joanna Lange, Arnthór Aevarsson, Ehmke Pohl

**Affiliations:** 1 Department of Chemistry, Durham University, Lower Mountjoy, Durham, County Durham, United Kingdom; 2 Bio-Prodict, Nijmegen, The Netherlands; 3 Matis ohf, Reykjavik, Iceland; 4 Department of Biosciences, Durham University, Upper Mountjoy, Durham, County Durham, United Kingdom

**Keywords:** metagenomics, thermophile, cysteine protease, protein crystallization, structure-function, structural biology, X-ray crystallography, zinc, protein evolution, Candidate Phyla Radiation, CPR, Candidate Phyla Radiation, TSA, Thermal shift analysis

## Abstract

The Candidate Phyla Radiation is a recently uncovered and vast expansion of the bacterial domain of life, made up of largely uncharacterized phyla that lack isolated representatives. This unexplored territory of genetic diversity presents an abundance of novel proteins with potential applications in the life-science sectors. Here, we present the structural and functional elucidation of CPR-C4, a hypothetical protein from the genome of a thermophilic Candidate Phyla Radiation organism, identified through metagenomic sequencing. Our analyses revealed that CPR-C4 is a member of a family of highly conserved proteins within the Candidate Phyla Radiation. The function of CPR-C4 as a cysteine protease was predicted through remote structural similarity to the *Homo sapiens* vasohibins and subsequently confirmed experimentally with fluorescence-based activity assays. Furthermore, detailed structural and sequence alignment analysis enabled identification of a noncanonical cysteine-histidine-leucine(carbonyl) catalytic triad. The unexpected structural and functional similarities between CPR-C4 and the human vasohibins suggest an evolutionary relationship undetectable at the sequence level alone.

The Candidate Phyla Radiation (CPR) is a vast collection of phyla and superphyla in the bacterial domain of life that currently lack isolated representatives (1, 2). There is on-going debate over the expanse of this radiation, with some predicting that the CPR comprises half of all bacterial diversity within the tree of life (3, 4, 5). As such, these candidate phyla have earned the nickname ‘microbial dark matter’, likely accounting for a significant portion of the Earth’s biomass but with largely unknown properties at present (6, 7).

While genome sizes and metabolic capabilities within a phylum are usually highly variant, organisms within the CPR have consistently small genomes (from 0.7 – 1.2 Mbp) and share a similarly limited set of biosynthetic pathways (2, 8, 9). It is likely that the limited metabolisms of the CPR and possibly a strict requirement for symbiotic lifestyles, are responsible for the difficulties associated with their cultivation. In addition, the ribosomal RNA sequences of CPR organisms have been reported to contain highly uncommon features, including encoded proteins and self-splicing introns (10). There is also substantial divergence in 16S ribosomal RNA sequences in CPR species. As these sequences are the key for uncovering new species by PCR techniques, it is estimated that at least half of the organisms sampled from the CPR would remain undetected in typical PCR surveys (10). These unusual properties point to atypical biology for a major part of the bacterial domain and have played a major part in the CPR remaining undetected for so long.

As well as improving our understanding of this newly uncovered yet expansive region of the tree of life, the exploration of novel gene products is the key for discovering enzymes that present new or unusual functions, as exemplified by the discovery of the CRISPR-Cas system (11). The hunt for genomes in extreme natural environments by the Virus-X consortium has unearthed and categorized over 50 million genes using a metagenomic approach (12). This method involves sequencing all of the genetic information isolated from a natural environment, including that which has previously evaded detection (8). The use of metagenomics techniques exploiting next generation sequencing technologies eliminates the need for cultivation and has made the genetic content of the CPR much more accessible for exploration (3, 4, 13, 14). In the Virus-X project, the natural systems explored include Icelandic geothermal hot springs and the Loki’s Castle hydrothermal vent system on the Mid-Atlantic ridge between Greenland and Norway. The bioprospecting activities concentrated on genomes from such extreme habitats as sources of novel enzymes with desirable properties, including tolerance to extremes of temperature, pH, and salt concentration, and high innovation potential for applications in the life sciences industry, such as biotechnology and pharmaceuticals. Particular focus was given to hypothetical or ‘unknown’ gene products with undetermined properties and potentially new functionalities. The functions of these hypothetical proteins are impossible to predict from nucleotide sequence alone due to sequence divergence and a lack of detectable homology. As such, structure determination offers a clearer insight into potential function and understanding of protein families within the CPR. Structure-based multiple sequence alignments consequently enable direct comparisons that can uncover structural similarities between proteins in spite of low sequence identity.

This study focusses on one predicted gene product from the genome of the first identified thermophilic CPR bacterium, discovered in an Icelandic hot spring. This gene product was denoted CPR-C4 and annotated as a hypothetical protein, with no characterized biological function. The lack of sequence identity to any proteins of known function in public databases, along with the identification of hundreds of related hypothetical protein sequences, led to the categorization of CPR-C4 as a high-priority target for structural determination with a potentially novel function or mechanism. In this article, we present the X-ray crystal structure of CPR-C4 solved by multiple-wavelength anomalous diffraction techniques at 2.60 Å and determined in three crystal forms to a maximum resolution of 2.25 Å. Subsequent deep sequence and structural analyses revealed an unexpected similarity to the *Homo sapiens* tubulin-tyrosine carboxypeptidases despite no significant sequence identity. This enabled the functional annotation of CPR-C4 as a protease with an unusual cysteine-histidine-leucine(carbonyl) catalytic triad and points toward an intriguing evolutionary relationship in this cysteine protease superfamily.

## Results

### Target identification and sequence analysis

The CPR-C4 gene was identified through metagenomic sequencing of a water sample from a terrestrial hot-spring in Iceland, with conditions of 75 °C and pH 6.0. The gene is one of 442,373 sequenced genes in the total metagenome from this sample, forming part of a contig of over 400 kbp in length and comprised of 432 genes. This contig was classified as belonging to a bacterial organism within the CPR through taxonomic annotation (Fig. S1). BLAST (15) searches enabled assignment of known or predicted functions to only 30% of the contig gene products. CPR-C4 was part of the remaining 70% for which it was not possible to identify a putative function through sequence alone, resulting in its annotation as a hypothetical protein. When classifying the genes based on the phylum classification level of their most significant BLAST (15) hit, we found that almost three quarters were categorized as either *Candidatus Adlerbacteria* or some unclassified bacterial phylum, with the remainder mostly from other *‘Candidatus’* phyla.

**Figure 1:**
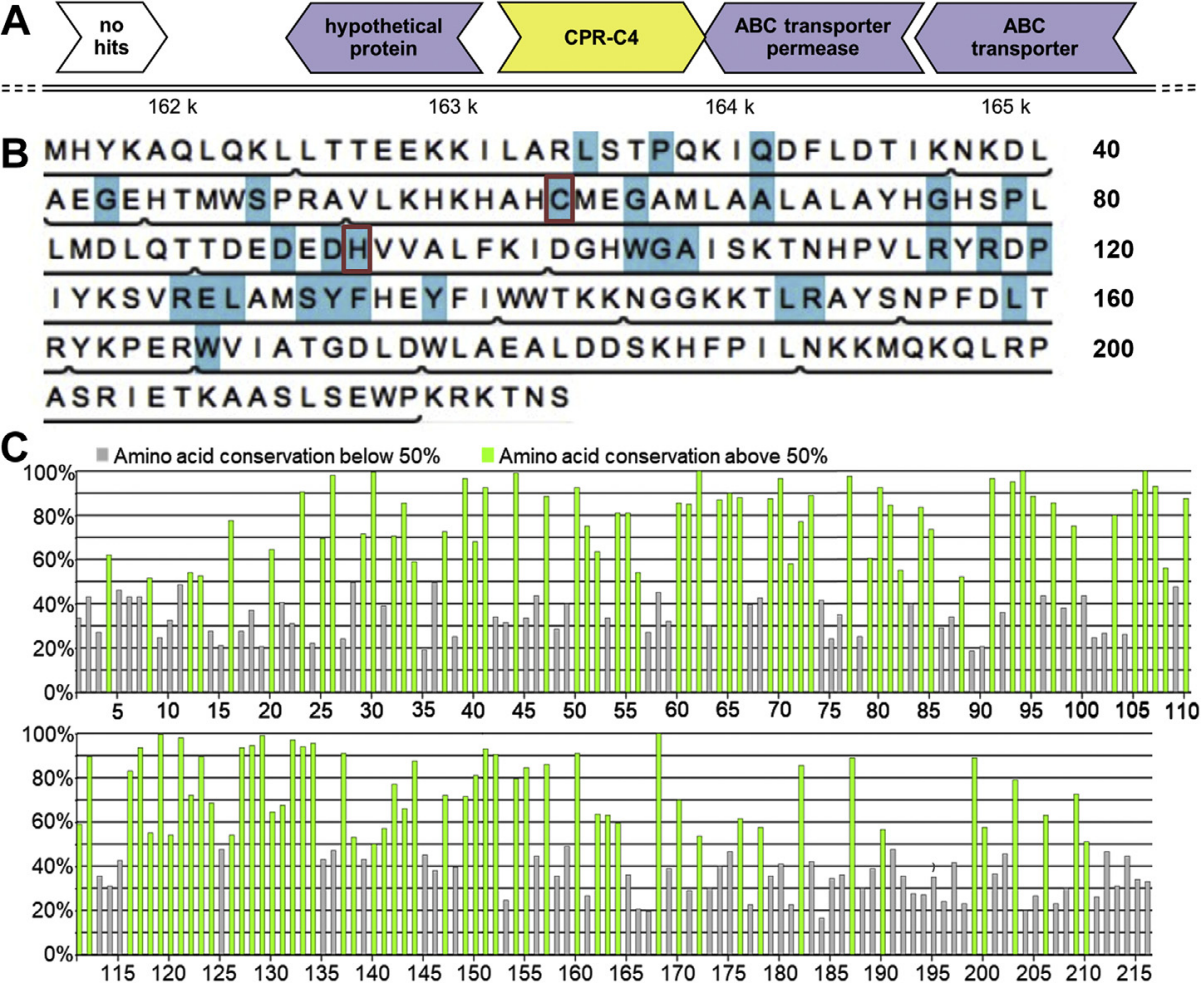
**CPR-C4 gene location and residue conservation.***A*, region of the contig containing the CPR-C4 gene and its neighbors, with predicted functions of gene products stated where annotation has been possible. Predicted genes are depicted as *arrows* following the strand direction of transcription and are color coded by the phylum classification level of their most significant BLAST (15) hit: *yellow* = unclassified bacteria; *pale purple* = *Candidatus Adlerbacteria*; *white* = unknown. *B*, amino acid sequence of the CPR-C4 protein, with residues conserved in ≥90% of instances in the CP01A subfamily shown in *blue*. Residues falling within the subfamily core region are underlined. 99.9% conserved residues C61 and H93 (following CPR-C4 numbering) are boxed in *red*. *C*, percentage conservation of residue identities within the CP01A subfamily core region generated using the 3DM alignment statistics tool following 3DM numbering. *Green bars* represent conservation of over 50% at that residue position; *gray bars* show conservation less than 50% at that position. The 99.9% conserved C61 and H93 (CPR-C4 numbering) are at positions 62 and 94, respectively. CPR, Candidate Phyla Radiation.

The CPR-C4 gene product was of particular interest as a hypothetical protein, with unknown function but showing extended conservation across CPR and non-CPR bacteria. Although searches of publicly available databases did not lead to assignment of any enzymatic domains or uncover potential structural homologs, BLAST (15) identified hundreds of hypothetical proteins with significant sequence similarity to CPR-C4 (over 400 with an E value < 1e^−50^). The CPR-C4 gene and its neighbors are shown in Figure 1*A*, which indicates that CPR-C4 is transcribed in the opposite direction to its flanking genes. Genes encoding proteins for ABC transport systems were identified adjacent to CPR-C4, though similar genes were not identified in proximity to CPR-C4 homologs in *Candidatus Kaiserbacteria* and *Candidatus Adlerbacteria* genomes. The gene position therefore does not offer any potential function for CPR-C4, though the sequence analysis points to an important and conserved protein in the CPR and beyond.

Deeper sequence analysis with the 3DM software (16) generated a subfamily for CPR-C4, denoted CP01A, containing 794 protein sequences. Twenty five of these, including CPR-C4, were identified by the Virus-X consortium. Of the 221 residues in the CPR-C4 sequence, the first 215 were found to fall within a conserved ‘core region’ in the subfamily (Fig. 1*B*). The extent of conservation in this core region is shown in Figure 1*C*. Relative to a highly conserved center, residues at the N and C termini are increasingly variable across the CP01A subfamily. The overall conservation of sequence in the subfamily is stark, with over half (110/216) of the residue identities conserved in ≥50% of the 794 subfamily sequences and 15% (32/216) of residues conserved in ≥90% of instances. This analysis highlights that CPR-C4 is part of a family of proteins with significant sequence conservation but unknown function; however, several residues can be identified that are almost entirely conserved and, as such, can be considered important for function. For example, C61 and H93 (CPR-C4 sequence numbering, Fig. 1*B*), residue types often involved in enzymatic activities, are 99.9% conserved in the subfamily. Structure determination was necessary to uncover the overall fold, analyze residue interactions, and assess the influence of these highly conserved residues, to uncover protein function.

### Protein production and biophysical characterization

Preliminary small-scale experiments confirmed that the ‘hypothetical’ CPR-C4 protein could be induced to overexpress in an *E. coli* host, though initial expression and purification experiments resulted in low protein yields, producing less than 0.5 mg of soluble protein per l of culture. It was also noticeable that CPR-C4 bound tightly to the immobilized Ni^2+^ column during purification, requiring an unusually high concentration of imidazole (>500 mM) to elute. SDS-PAGE analysis and electrospray ionization mass spectrometry confirmed the identity of CPR-C4 based on expected molecular weight (27,121 Da, Fig. S2), and size-exclusion chromatography identified that the protein exists in dimeric form (Fig. S3).

### Thermal shift analysis

Thermostability is a highly desirable feature of proteins for biotechnology applications (17, 18, 19). Thermal shift analysis (TSA) can be used to assess the intrinsic thermotolerance of proteins, as well as identify additives and buffer systems that can improve thermostability (20, 21). TSA was conducted to establish stabilizing conditions for CPR-C4 in order to improve yields and prevent precipitation, which made room temperature purification challenging. Purification with a phosphate-based buffer system, in place of the Hepes buffer used in initial experiments, increased the amount of soluble protein obtained after purification, reflected by a stabilization of approximately 4 °C in phosphate relative to Hepes in TSA with the Durham pH Screen.

**Figure 2:**
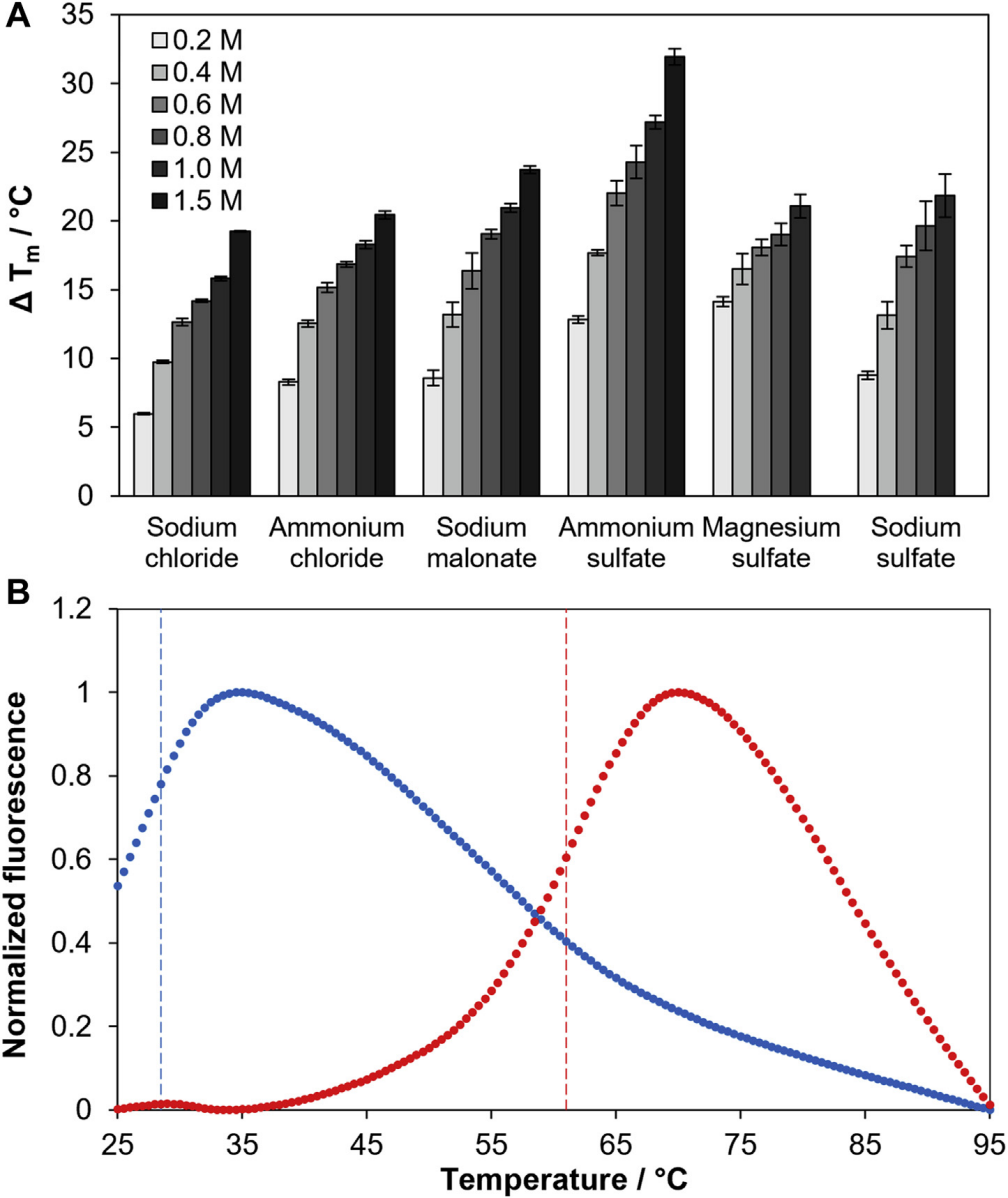
**Thermal shift analysis of CPR-C4.***A*, results from TSA of CPR-C4 with the Durham Salt Screen showing changes in melt temperature, T_m_, upon addition of stabilizing salts at increasing concentrations (n = 3, ± st. dev.). *B*, normalized fluorescence intensities at 590 nm for TSA of CPR-C4 in water (*blue*) and with addition of 1 mM ZnCl_2_ (*red*). Raw experimental data are shown as individual points; *dashed vertical lines* indicate the calculated T_m_ values of CPR-C4 in water (*blue*) and with addition of 1 mM ZnCl_2_ (*red*) (20). CPR, Candidate Phyla Radiation; TSA, Thermal shift analysis.

The TSA results of CPR-C4 with the Durham Salt Screen are represented in Figure 2. The majority of additives screened resulted in some level of stabilization relative to the reference melt temperature, T_m_, in water. The protein is markedly stabilized by a range of inorganic salts, including several sodium and ammonium salts that caused a ≥20 °C increase in T_m_ at the highest concentration tested (Fig. 2*A*). This consistent increase in thermotolerance shows that CPR-C4 is stabilized by high salt concentrations, with negligible preference for chemical composition.

While CPR-C4 is not intrinsically thermostable, with an unexpectedly low reference T_m_ of only 29.1 ± 0.5 °C (n = 6), it was hugely stabilized by the addition of divalent metal cations. This is most dramatic with the addition of ZnCl_2_, as demonstrated by an increase in T_m_ of over 30 °C (T_m_ 60.9 ± 0.2 °C, n = 3, Fig. 2*B*). Consequently, protein yields were improved more than 20-fold with the addition of 100 μM ZnCl_2_ to expression media, producing >10 mg of purified protein per l of culture. The increase in soluble protein yield resulting from these small alterations to expression and purification procedures after TSA was critical for downstream assays and demonstrates the stabilizing effect of Zn^2+^ on CPR-C4.

### Structure determination

The CPR-C4 protein, including an N-terminal His_6_-tag (Fig. S4), crystallized in two space groups with different crystal morphologies but similar resolution limits (Table 1). X-ray fluorescence scans identified an unambiguous Zn signal from crystal form 1, and the subsequent X-ray absorption edge scan (Fig. S5) was used to select appropriate wavelengths for data collection, optimizing the collection of an anomalous Zn signal for multiple wavelength anomalous diffraction phasing (22). It should be noted that these crystals were grown using protein expressed without the addition of Zn to media. The crystals diffracted to a maximum resolution of 2.60 Å in P3_2_21, with two protein chains generating the functional dimer showing two-fold rotational noncrystallographic symmetry and two Zn^2+^ ions in the asymmetric unit. The structure solution was nontrivial: model building after Zn substructure solution with SHELXD (23) required data processed with autoPROC using STARANISO (24, 25). The density for chain A, which was built first, was notably better defined so that even the side chains of the His_6_-tag were interpretable. Crystal form 2 diffracted to a resolution of 2.68 Å in space group I222, with one polypeptide chain per asymmetric unit, and was solved by molecular replacement with the model from crystal form 1. To assess the effect of the non-native His_6_-tag, crystallization was also subsequently conducted using protein without the tag (Fig. S4*D*), resulting in a third crystal form in space group P2_1_2_1_2_1_ and improved resolution (2.25 Å), with one dimer per asymmetric unit. Interface and assembly analysis of the form 1 CPR-C4 dimer with PISA (26) showed an interface area of 1669.1 Å^2^ (15%) and a binding energy of ^−^26.2 kcal mol^−1^. This strongly suggests that this interface is part of the biological assembly of the protein and, alongside size exclusion analysis (Fig. S3), confirms that the protein exists naturally in dimeric form.

**Table 1:** Crystallographic data collection and refinement statistics

Parameter	Form 1	Form 2	Form 3
Beamline	DLS I03	DLS I24	DLS I04
Wavelength (Å)	1.2824	1.2819	0.979
Resolution range (Å)	48.26–2.60 (2.71–2.60)	67.40–2.68 (2.81–2.68)	44.26–2.25 (2.32–2.25)
Space group	P3_2_21	I222	P2_1_2_1_2_1_
Unit cell dimensions (Å), (°)	123.39, 123.39, 96.53, 90, 90, 120	56.31, 79.73, 126.20, 90, 90, 90	44.78, 77.55, 161.72, 90, 90, 90
No. of reflections	224,888 (26,437)	79,870 (8047)	337,006 (16,676)
Unique reflections	26,494 (3143)	8223 (995)	27,119 (2058)
Multiplicity	8.5 (8.4)	9.7 (8.1)	12.4 (8.1)
Completeness (%)	99.8 (99.2)	98.8 (92.7)	98.1 (85.3)
⟨I/σ(I)⟩	14.4 (0.7)	7.4 (1.4)	17.1 (1.1)
Wilson B-factor (Å^2^)	75.3	45.3	55.9
R_merge_	0.094 (4.086)	0.453 (6.072)	0.085 (1.770)
R_pim_	0.050 (2.198)	0.153 (2.225)	0.025 (0.666)
CC_1/2_	1.000 (0.368)	0.972 (0.055)	0.999 (0.360)
R_work_	0.234	0.238	0.179
R_free_	0.285	0.259	0.225
Number of atoms	3412	1671	7223
Number of protein residues	435	211	433
RMSD bond lengths (Å)	0.0060	0.0061	0.0076
RMSD bond angles (°)	1.418	1.406	1.442
Ramachandran plot favored/allowed/outliers	409/18/0	197/9/1	407/19/0
Average B factor, overall (Å^2^)	109.0	51.0	61.0
PDB accession codes	7OB6	7OB7	7PJO

Values in parentheses are for the highest resolution shell.

**Figure 3:**
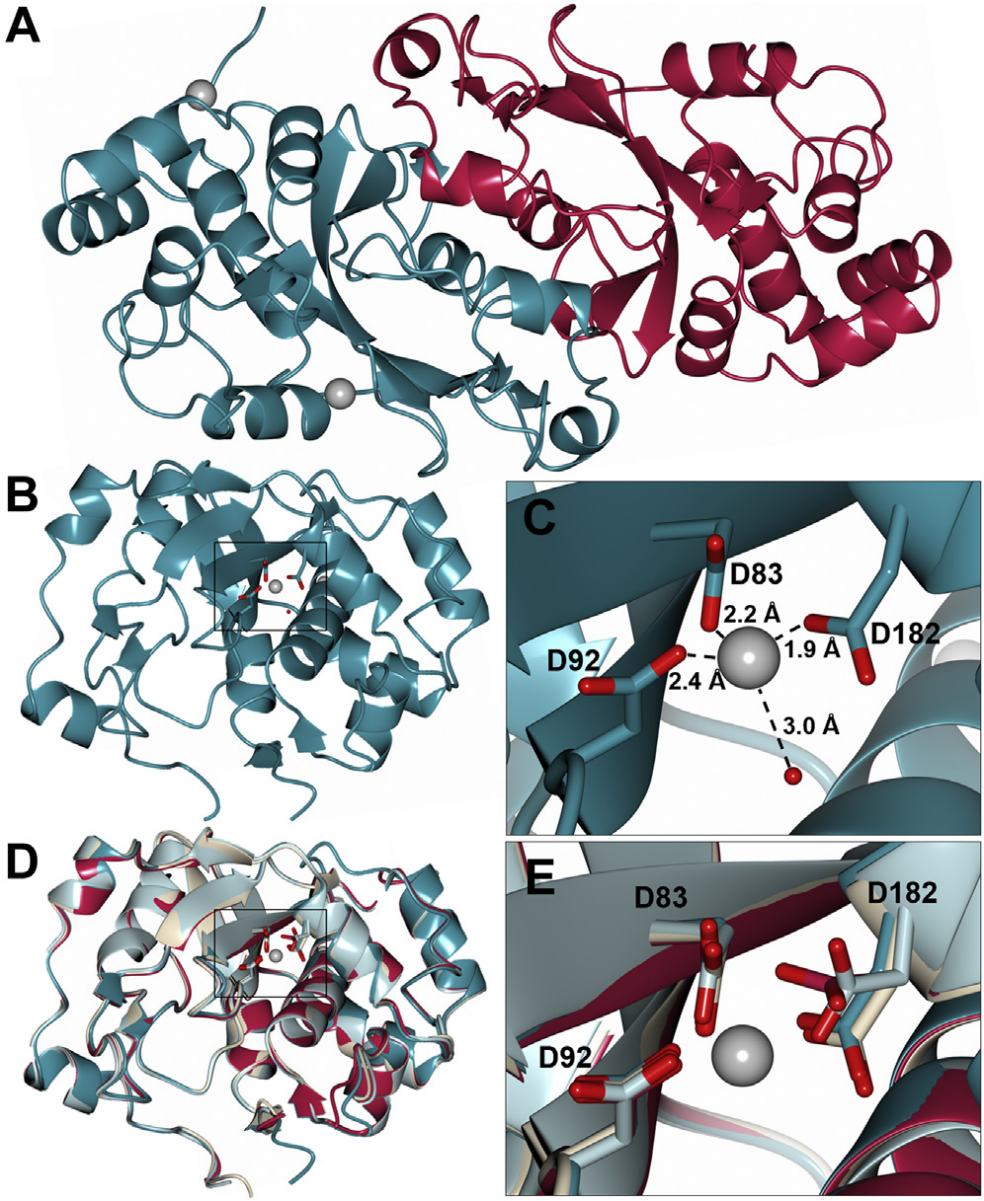
**Crystal structure of CPR-C4.***A*, ribbon diagram of the CPR-C4 dimer (crystal form 1) showing the two molecules in the asymmetric unit perpendicular to the rotational noncrystallographic symmetry axis; chain A is shown in *teal*, chain B in *crimson*; Zn^2+^ ions are shown as *gray spheres* with 1.0 van der Waals radii. *B*, CPR-C4 monomer (form 1 chain A) orientated to highlight the position of the tri-aspartate Zn^2+^-binding site, indicated with a *black box*. The main chain is shown with *ribbon* representation; aspartate side chains are shown with *cylinder* representation, O atoms in *red*; Zn^2+^ ions are shown as *gray spheres* with 0.5 van der Waals radii; the coordinated water molecule is shown as a *red sphere*. *C*, close-up view of the Zn^2+^-binding site as indicated in (*B*), showing tetrahedral coordination to three aspartate side chains and a water molecule; distances in Å are indicated with *dashed lines*; aspartate side chains are shown with *cylinder* representation, O atoms in *red*; the Zn^2+^ ions are shown as *gray spheres* with 0.5 van der Waals radii; the coordinated water molecule is shown as a *red sphere*. *D*, least-squares superposition of CPR-C4 chains in the same orientation as (*B*): form 1 chain A shown in *teal*, form 1 chain B in *crimson*, form 2 in *light blue*, and form 3 chain A in white; Zn^2+^ ions found in form 1 chain A only have been omitted for clarity. *E*, least-squares superposition of the tri-aspartate Zn^2+^ site across the CPR-C4 chains colored as in (D); Zn^2+^ ions from form 1 chain A are shown as *gray spheres* with 0.5 van der Waals radii. CPR, Candidate Phyla Radiation.

In all crystal forms, the structure of CPR-C4 is a homodimer displaying a mixed αβ fold, with a central curved antiparallel β-sheet surrounded by short α-helices connected by flexible loops (Fig. 3*A*). Surprisingly, the two Zn^2+^ ions were only present in crystal form 1 and were both found to interact with the same chain (chain A, Fig. 3*B*), in a tetrahedral geometry, as is most common for Zn^2+^ coordination (27). The first is coordinated by three aspartate side chains (D83, D92, D182) and a water molecule (Fig. 3*C*), and the second is coordinated by two histidine residues from the N-terminal His_6_-tag, another histidine (H75), and a glutamate residue (E179) from a neighboring symmetry mate, forming a crystal contact.

**Figure 4:**
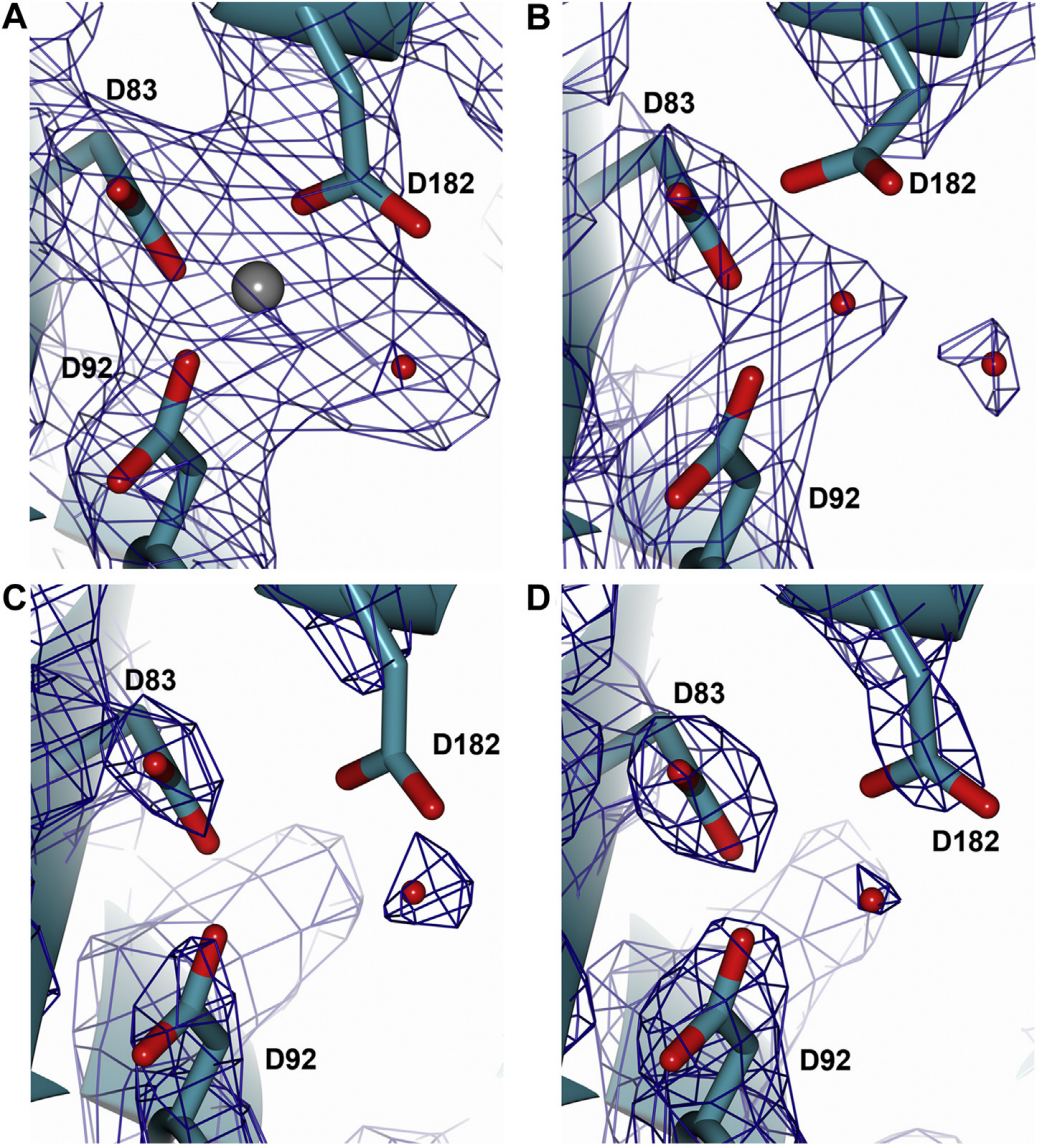
**Comparison of electron density at the tri-aspartate Zn**^**2+**^**-binding site between the CPR-C4 crystal forms.** In all panels, the main chain is shown with *ball* and *stick* representation in *teal*, O atoms in *red*, N atoms in *blue*, S atoms in *yellow*; water molecules are shown as *red spheres*. The 2|F_o_|-|F_c_| map is shown in *gray* with *cylinder* representation at contour level σ = 1.5. This high sigma was chosen to make clear the position of Zn^2+^ and coordinating residues in (A). At σ = 1.0, D182 is well fitted in density across all three crystal forms. *A*, electron density for crystal form 1 chain A showing the location of bound Zn^2+^, depicted as a *gray sphere*, with coordinating water molecule and aspartate residues. *B*, electron density for form 1 chain B. *C*, electron density for form 2. *D*, electron density for form 3 chain A. CPR, Candidate Phyla Radiation.

The three structures in the different crystal forms superimpose very well, with no significant deviation (Fig. 3*D* and Table S1). The two chains of form 3 are very closely aligned, with a RMSD of only 0.27 Å. The two chains of form 1 are less closely aligned (RMSD 0.52 Å), likely due to slight differences resulting from Zn^2+^ binding to one chain. While form 2 has a single chain in the asymmetric unit, a symmetry dimer is observed with RMSD values of 0.37 Å and 0.31 Å against the form 1 and form 3 noncrystallographic dimers, respectively (Fig. S6). Despite the lack of Zn^2+^ in crystal forms 2 and 3, the key aspartate residues superimpose well across all three structures (Fig. 3*E*) and the geometry of the binding site is maintained in each structure. Figure 4 shows a comparison of electron density at this tri-aspartate site across the CPR-C4 crystal structures, demonstrating that Zn^2+^ is only present in crystal form 1, with no other metal cations binding in its absence. These results show that, although Zn^2+^ dramatically stabilizes the protein, its presence does not affect the overall structure and the protein adopts its native conformation even in the absence of Zn^2+^.

The inherent inability to cultivate the source organism makes it difficult to confirm that Zn^2+^ is the native metal of the CPR-C4 tri-aspartate binding site. However, the absence of other metal cations in the zinc-free crystal structures implies this is not a promiscuous site, and the tetrahedral ligand arrangement is strongly preferable for Zn^2+^ binding over other transition metal cations or the sodium and potassium present in the buffers used (28). Zn^2+^ was also incorporated into the protein without being supplemented in expression media or buffers, and TSA showed vast protein stabilization upon the addition of Zn^2+^. This evidence all supports the presence of an intrinsic Zn^2+^-binding site in CPR-C4.

### Bioinformatic structural analysis for determination of function

**Figure 5:**
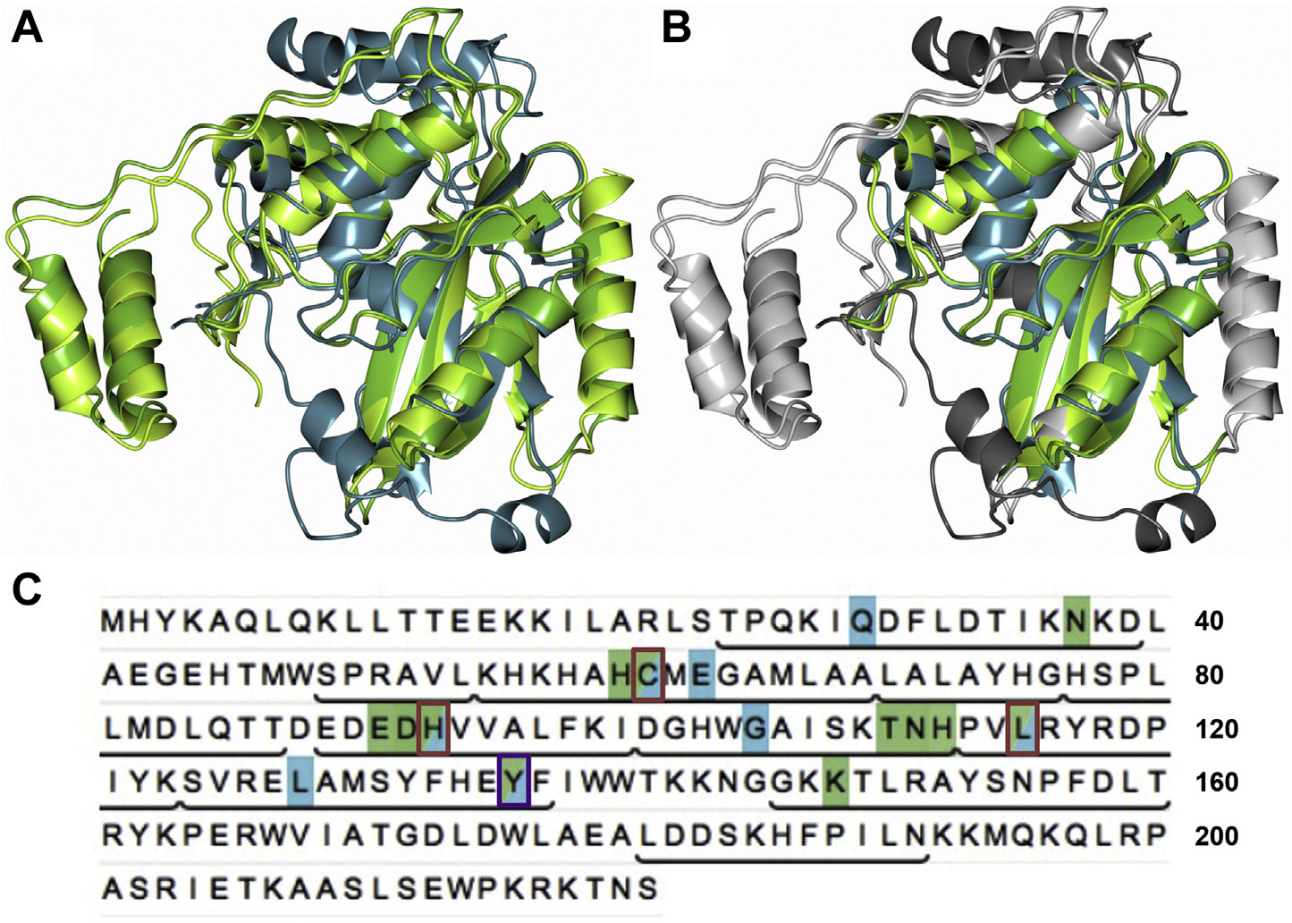
**Sequence and structural analysis of CPR-C4 with 3DM.***A*, ribbon diagram showing the structural alignment of CPR-C4 (form 1 chain A, *teal*) with *Homo sapiens* VASH1 (6J8F chain B, *green*, RMSD 1.98 Å) and VASH2 (6J4P chain A, *yellow green*, RMSD 1.92 Å) through least-squares superposition. *B*, the 3DM core region, sharing significant structural similarity across CPR-C4 and human VASH1/2, is shown in the same color representation as (*A*); the remaining ‘variable regions’ are shown in *dark gray* for CPR-C4 and in *light gray* for VASH1/2. *C*, amino acid sequence of CPR-C4, with the 3DM core region underlined. Residues conserved in ≥95% of 3DM superfamily members are shown in *blue*; residues that form ≥8 ligand contacts in the superfamily are shown in *green*. The three residues boxed in *red* form the conserved catalytic triad (C61, H93, L115, following CPR-C4 sequence numbering). The reside boxed in *purple* is the conserved tyrosine involved in Leu(carbonyl) positioning in VASH1/2 (30). CPR, Candidate Phyla Radiation.

In order to determine the function of the novel CPR-C4 protein, 3DM (16) analysis was conducted to identify structural homologs for detailed comparison. Remarkably, two proteins from *H. sapiens*, tubulin tyrosine carboxypeptidases 1 and 2 (also known as vasohibins VASH1 and VASH2, respectively), were identified as sharing the core mixed αβ fold of CPR-C4 (Fig. 5*A*). These carboxypeptidases, bound to the α-helical small vasohibin binding protein, cleave the C-terminal tyrosine residue of α-tubulin as part of microtubule regulation, utilizing a noncanonical Cys-His-Leu(carbonyl) catalytic triad (29, 30, 31, 32). These human isoforms are part of the transglutaminase-like cysteine protease superfamily and share a sequence identity of 87.8% with each other, though only show 17.6% (VASH1) and 15.4% (VASH2) sequence identity to CPR-C4.

To further investigate the evolutionary relationship between these proteins, the 3DM system for CPR-C4 was subsequently extended to include the two additional subfamilies of the newly identified human VASH1/2 structural homologs. These subfamilies were denoted 6J8FB and 6J4PA after their template structure Protein Data Bank accession codes (29). The resulting 3DM superfamily system contains 657 and 817 sequences homologous to the human VASH1 and VASH2 proteins, respectively.

**Figure 6:**
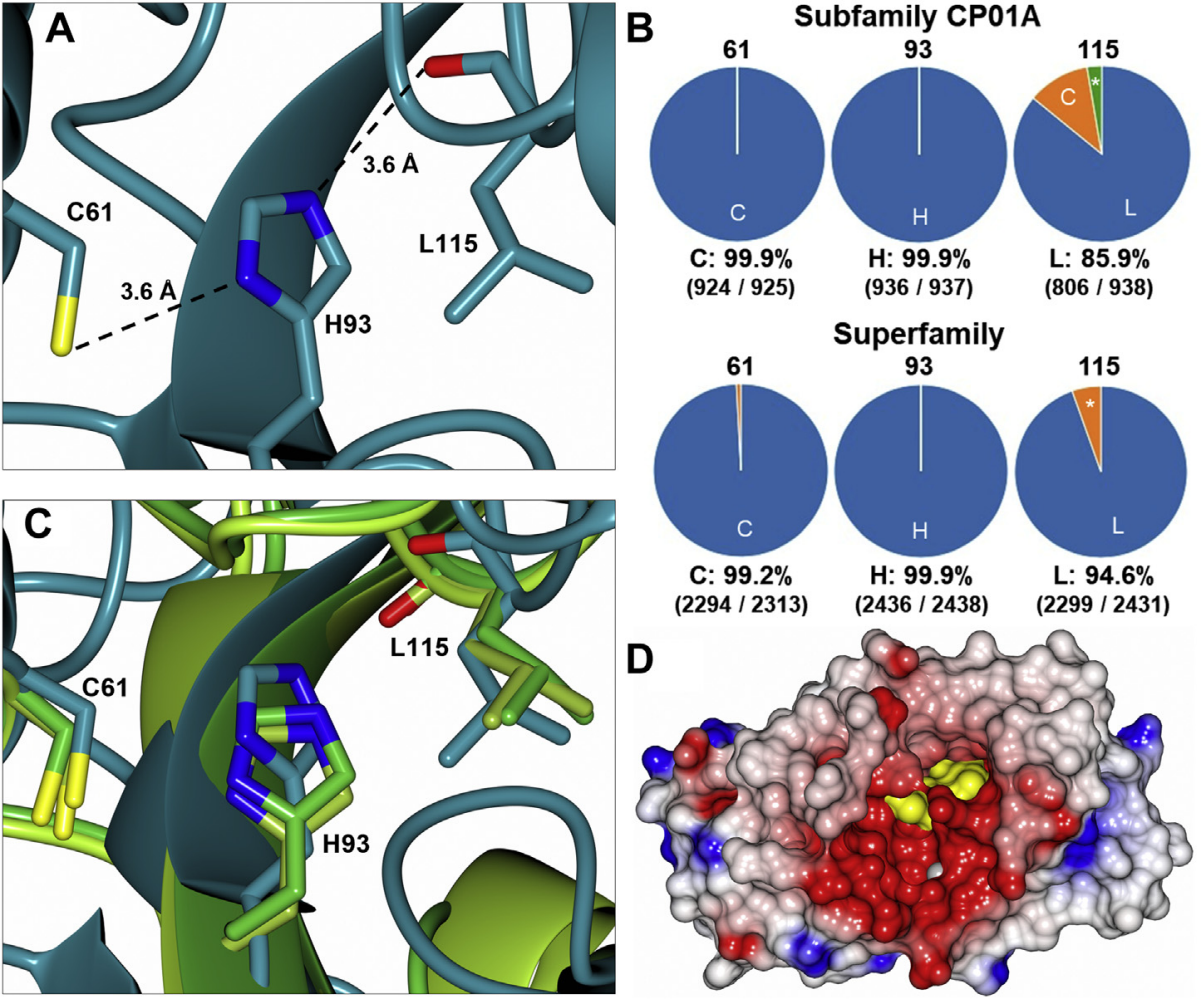
**Analysis of the CPR-C4 catalytic triad.***A*, close-up view of the catalytic triad residues in the CPR-C4 crystal structure (form 1 chain A). The main chain is shown with *ribbon* representation in *teal*; side chains of the catalytic triad residues and the backbone carbonyl of L115 are shown with *stick* representation, O atom in *red*, N atoms in *blue*, S atom in *yellow*; interatomic distances in Å are indicated with *dashed lines*. The average B-factors for these residues are 92.9 Å^2^, 89.4 Å^2^, and 84.9 Å^2^ for C61, H93, and L115, respectively. *B*, conservation of the catalytic triad residue identities across the CP01A subfamily and the extended superfamily comprising the CP01A, 6J4PA, and 6J8FB subfamilies; ∗ indicates all other residue identities. *C*, catalytic triad residues in CPR-C4 (form 1 chain A, *teal*) aligned with equivalent 3D residues from human VASH1 (6J8F chain B, *green*) and VASH2 (6J4P chain A, *yellow green*) through least-squares superposition; the main chain is shown with *ribbon* representation; catalytic triad side chains and the backbone carbonyl group of L115 are shown with *cylinder* representation, O atoms in *red*, N atoms in *blue*, S atoms in *yellow*. *D*, electrostatic surface representation of CPR-C4 (form 1 chain A) showing the proposed substrate-binding pocket of CPR-C4, lined with positively charged residues; *red* shows positive potential, *blue* shows negative potential; the surfaces of the catalytic triad residues are shown in *yellow*; Zn^2+^ ion represented as a *gray sphere* with 1.0 van der Waals radius. CPR, Candidate Phyla Radiation.

The 3DM alignment tool (16) was then used to identify highly conserved residues in the superfamily as well as residues known to form ligand contacts in structures collated in the 3DM system. One hundred thirty of 221 residues in the CPR-C4 sequence fall within the structurally conserved core region for the extended superfamily, as shown in Figure 5, *B* and *C*. Also within this region is the known substrate-binding site of the vasohibins (29): of the 17 residue positions in VASH1/2 identified to form ligand contacts, 15 positions were found in the core region shared with CPR-C4. Only five of the 15 residue identities were conserved in the CPR-C4 sequence; however, three of these importantly comprise the known catalytic triad of VASH1/2 required for protease activity: C61, H93, and L115 (following the CPR-C4 sequence) (Fig. 6*A*). This unconventional catalytic triad is highly conserved throughout all three subfamilies (Fig. 6*B*), and the residues structurally superpose well across the three template structures (Fig. 6*C*). C61 and H93 are almost entirely conserved in both the CP01A subfamily and the extended 3DM superfamily, and leucine is the dominant residue identity at position 115. C61, H93, and L115 also form nine, 10, and eight ligand contacts in the superfamily, respectively, highlighting their importance for ligand binding in VASH1/2.

The occurrence of leucine makes this an atypical catalytic triad for a cysteine protease. Based on the mechanism suggested for the vasohibins, it was proposed that the backbone carbonyl of L115 in CPR-C4 forms a hydrogen bond (H-bond) with the imidazole ring of H93, which in turn activates the cysteine thiol. The tyrosine residue known to help position the key leucine carbonyl for H-bonding with His in the human vasohibins is also present in CPR-C4, with 92.5% conservation in the CP01A subfamily and 95.8% conservation in the extended superfamily (Fig. S7). Intriguingly, a serine residue (S108) was also identified in close proximity to H93, at a distance appropriate for H-bonding (2.8 Å), which could play an additional role in activation for catalysis. Nonetheless, Ser is only present in 41.9% of CP01A subfamily sequences, with Ala in over 50% (473/938) and Gly in all vasohibin family sequences. This serine residue is therefore unlikely to be crucial to function, though may play a role in some CP01A subfamily proteins.

While the conservation of the unusual catalytic triad in both sequence and structure is a crucial indicator of shared function, there are key differences between CPR-C4 and the vasohibins. Unlike VASH1/2, which are known to require a small binding protein, there is no indication that CPR-C4 requires a binding partner for catalysis. CPR-C4 is a homodimer and lacks the helices that are known to form intermolecular contacts between monomeric VASH1/2 and the small vasohibin binding protein. In the vasohibin structures, the conserved catalytic triad is also located deep in a negatively charged pocket, known to be the substrate binding pocket for α-tubulin (29, 32). In CPR-C4, the pocket containing the proposed C61-H93-L115 catalytic triad is formed of positively charged residues (Fig. 6*D*), implying a negatively charged substrate. This is in contrast to the positively charged α-tubulin substrate of VASH1/2, which is also crucially lacking in bacteria. The structure-based multiple sequence alignment analysis therefore points to CPR-C4 functioning as a cysteine protease, utilizing the same noncanonical catalytic triad as the VASH1/2 structural homologs, albeit with a very different substrate.

### Analysis of the Zn^2+^-binding site

**Figure 7:**
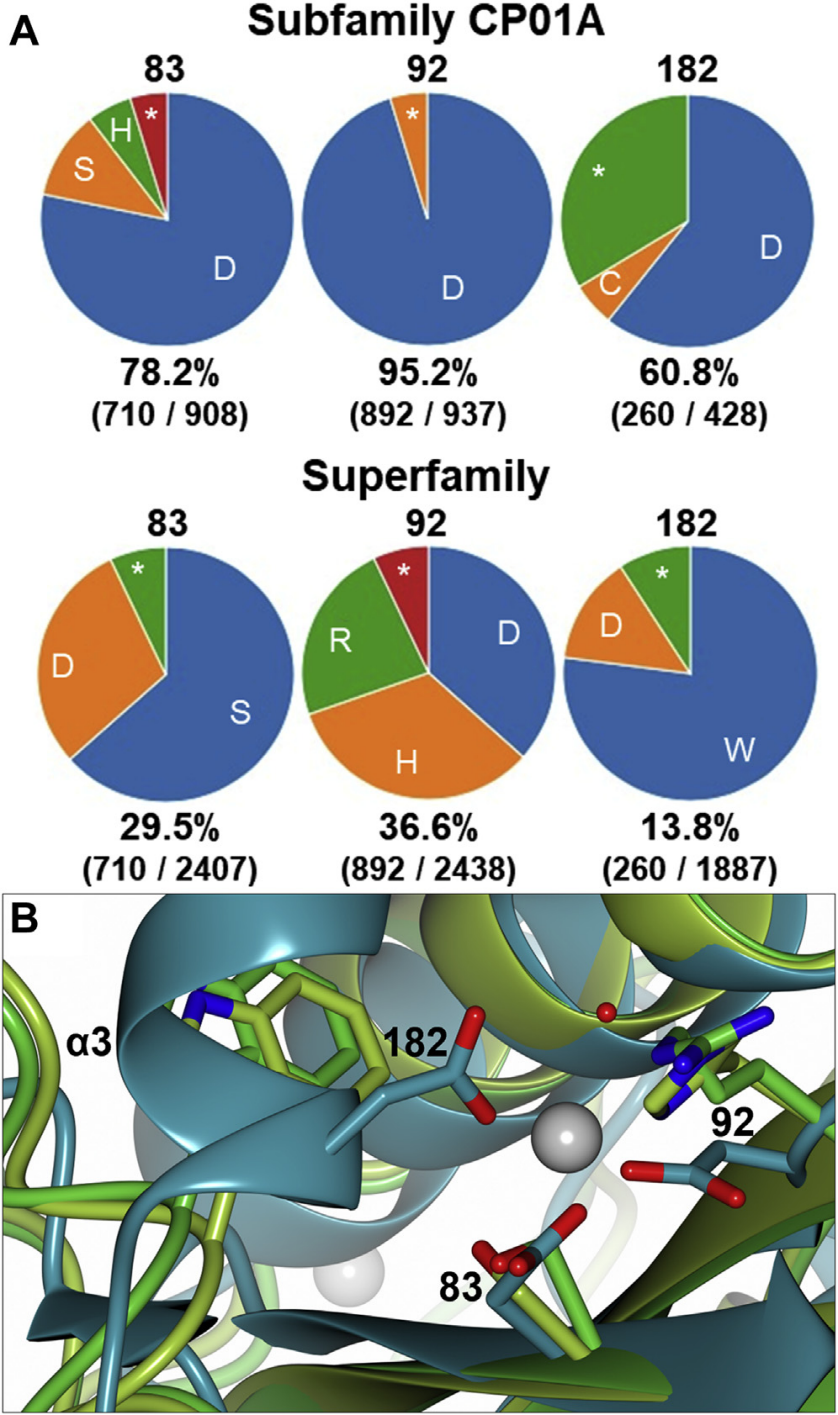
**Analysis of the CPR-C4 tri-aspartate Zn**^**2+**^**-binding site.***A*, conservation of equivalent residues of the CPR-C4 Zn^2+^-binding site across the CP01A subfamily and the extended superfamily containing the CP01A, 6J4PA, and 6J8FB subfamilies; ∗ indicates all other residue identities. *B*, Zn^2+^-binding site from the CPR-C4 structure (form 1 chain A, *teal*) aligned with equivalent residues in human VASH1 (6J8F chain B, *green*) and VASH2 (6J4P chain A, *yellow green*) through least-squares superposition; the main chain is shown with *ribbon* representation; residue side chains are shown with *cylinder* representation, O atoms in *red*, N atoms in *blue*; Zn^2+^ ions are shown as a *gray spheres* with 0.5 van der Waals radii and the coordinated water molecule is shown as a *red sphere* (note: these are only present in the CPR-C4 form 1 chain A structure). CPR, Candidate Phyla Radiation,

To evaluate the role of Zn^2+^, the extent of aspartate conservation at the binding site residue positions was assessed across the CP01A subfamily and the wider superfamily (Fig. 7*A*). All three positions are in the core structural region conserved between the subfamilies: following the CPR-C4 sequence, these positions are denoted 83, 92, and 182. There is considerable conservation in the CP01A subfamily, with aspartate being the predominant residue at all three positions. However, less than half of the sequences contain a residue aligned at position 182. The tri-aspartate Zn^2+^-binding site is therefore not a requirement for proteins in the CP01A subfamily.

The conservation of aspartate seen in the CP01A subfamily also does not extend to the wider superfamily. All instances of aspartate at these positions in the superfamily occur in sequences from the CP01A subfamily, with none in 6J8FB or 6J4PA subfamily sequences. At position 83, the predominant residue in the superfamily is serine, occurring in 63.4% of sequences. At position 92, there are similar proportions of aspartate, histidine, and arginine, and tryptophan is overwhelmingly the most common residue at position 182 (77.0%). Throughout the 6J4PA or 6J8FB subfamilies, there are also no residues known to form metal ion contacts. Residues 83, 92, and 182 in CPR-C4, and equivalent residues in VASH1/2, are superposed in Figure 7*B*. While 83 and 92 superpose well across the structures, the coordination of D182 to Zn^2+^ in CPR-C4 pulls the chain out of alignment with the vasohibin structures at the start of the α3 helix. This evidence collectively points to a lack of conservation of the CPR-C4 Zn^2+^-binding site in the vasohibin subfamilies.

### Protease activity of CPR-C4

**Figure 8:**
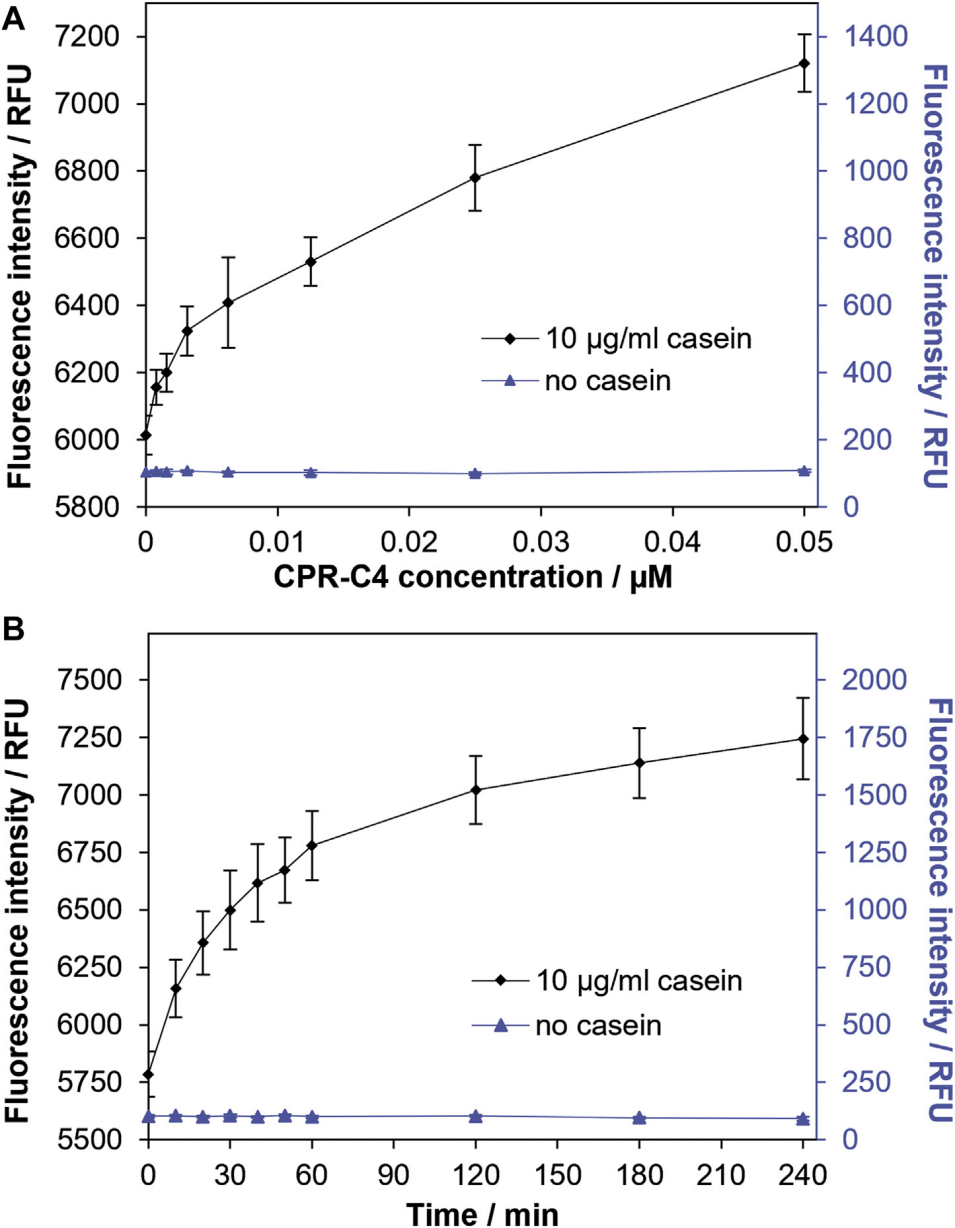
**Experimental analysis of CPR-C4 protease activity.***A*, protease activity data for increasing concentrations of CPR-C4 in the presence (n = 8, ± st. dev.) and absence (n = 3, ± st. dev.) of BODIPY-FL casein substrate, measured as fluorescence intensity in relative fluorescence units (RFU) after 3 h at 30 °C. *B*, time course of the increase in fluorescence intensity for 0.025 μM CPR-C4 in the presence (n = 8, ± st. dev.) and absence (n = 3, ± st. dev.) of BODIPY-FL casein substrate. CPR, Candidate Phyla Radiation.

The structure-based multiple sequence alignment analysis strongly implied that CPR-C4 is a protease, utilizing a cysteine-based catalytic triad to cleave an unidentified substrate. The enzyme is expected to be highly specific based on the unusual active site geometry and what is known of the VASH homologs. In order to confirm protease activity, biochemical assays were conducted using a casein derivative substrate labeled with green fluorescent BODIPY-FL dye (33), as a substitute for the unknown substrate. Highly purified protein (Fig. S2*A*) was used in these assays to minimize the likelihood of contamination. As shown in Figure 8*A*, the addition of increasing concentrations of CPR-C4 to a fixed amount of casein substrate resulted in a rise in fluorescence emission at 528 ± 20 nm after excitation at 485 ± 20 nm. Additions of CPR-C4 in the nM range (to a maximum of 0.05 μM) resulted in a significant increase in fluorescence relative to nonproteolytic controls. This indicates the presence of fluorescently labeled peptide fragments due to proteolytic cleavage of the BODIPY-FL casein substrate by CPR-C4. The time course shown in Figure 8*B* also shows that, at a fixed concentration of CPR-C4 (0.025 μM), the fluorescence intensity increases over time as the BODIPY-FL casein substrate is cleaved to fluorescent fragments by CPR-C4. This experimental data confirms that CPR-C4 shows protease activity, as was projected from the structure-based multiple sequence alignments.

### Phylogenetic analysis

In order to analyze the evolutionary relationship between CPR-C4 and the human VASH proteins in greater detail, a phylogenetic tree was generated from 92 protein sequences with significant similarity to CPR-C4 and VASH1/2 from a broad range of taxa (Fig. S8). These sequences are based on separate BLAST (15) searches of the CPR-C4 and VASH1/2 protein sequences using an E value cut-off of 0.05. After multiple sequence alignment with ClustalW (34), the phylogenetic tree was constructed using MEGA X (35) with maximum-likelihood methods. Bootstrap tests were also applied to assess the confidence.

The tree is divided almost perfectly in two, with sequences relating to human VASH1/2 congregating in the top half, grouped into animalia and fungi species in light and dark blue, and Plantae species in green. Sequences from *Firmicutes* bacteria were the only prokaryotic sequences identified with similarity to human VASH1/2 in the original BLAST (15) search. The lower half of the tree is comprised of bacterial (non-CPR and CPR in yellow and light yellow, respectively), archaeal (in red), and algal (in orange) protein sequences identified by BLAST (15) on CPR-C4. Importantly, no eukaryotic sequences were identified in this search. The taxa at the node linking these two groups of sequences only clustered together in 195/500 repeats (39%, Fig. S8). This implies a weak, if any, evolutionary relationship between CPR-C4 and the human vasohibins that is not identifiable by sequence analysis alone.

## Discussion

### Validation of the CPR-C4 ORF gene product

While a consistent feature of the CPR is small genomes, the CPR-C4 contig is significantly smaller (0.4 Mbp) than previously identified closed genomes from the CPR (0.7–1.2 Mbp) (2), suggesting it does not cover an entire genome. Due to their small size, CPR genomes could be expected to have a higher percentage of genes with known functions than usual, dominated by enzymes with near universal functions crucial for survival. However, less than one third of the genes comprising the CPR-C4 contig could be assigned predicted functions based on sequence analysis, compared to up to 70% of a typical newly sequenced bacterial genome (36). Since the unearthing of the CPR within the tree of life is still recent, it is unsurprising that such genomes remain poorly understood and contain large proportions of uncharacterized gene products. Many of the hypothetical proteins identified in the CP01A subfamily with high sequence similarity to CPR-C4 are also from candidate phyla bacteria, indicating that this protein is highly conserved and therefore important within the CPR.

### Thermostability

Considering that the source organism of CPR-C4 originates from a sample collected at 75 °C, it is perhaps surprising that the T_m_ of CPR-C4 without additives is so low (Fig. 2*B*). While it may be expected that proteins originating from thermophilic organisms would be intrinsically thermostable, this is not guaranteed. The protein environment *in vivo* is often responsible for a large amount of stabilization, including through molecular chaperones and compatible solutes (37). CPR-C4 was indeed stabilized by a wide variety of salts at high concentrations (Fig. 2*A*), which provides an insight into how thermotolerance is achieved in its natural environment.

### Role of Zn^2+^ in CPR-C4

Zn^2+^ ions in proteins can be structural or participate directly in enzyme function (38). The nature of the CPR-C4 Zn site is at first glance indicative of a catalytic over a structural Zn site, where the metal coordination sphere is often comprised of protein side chains only (39, 40). However, the arrangement and identities of the residues forming this metal binding site are unusual. In classic catalytic Zn metalloproteins, two of the protein ligands (L1 and L2) are typically in close proximity in the sequence, that is, one to three amino acids apart, with much greater variation in the distance of the third coordinating residue (L3) along the chain (38). In the case of CPR-C4, the first two ligands (D83) and L2 (D92) are separated by eight residues, and L3 (D182) is well removed from the first two ligands (90 residues). The tri-aspartate arrangement is also atypical, with histidine accounting for the majority of residues coordinated to Zn^2+^ in catalytic sites of Zn metalloproteins and cysteine for structural sites (36, 37). Considering the metal coordination alone, CPR-C4 therefore appears to contain an unusual but possible catalytic Zn^2+^ site of unknown function. However, since Zn^2+^ or other metal ions are not required for protease activity in the vasohibins, it is unlikely that the Zn^2+^ in CPR-C4 has a catalytic role or is a requirement for activity. The TSA experiments show that Zn^2+^ instead plays a stabilizing structural role, increasing the thermotolerance of the protein significantly.

### From structure to function

The novel crystal structure of CPR-C4 demonstrates the power of structure determination for unraveling the function of hypothetical proteins for which sequence alone is insufficient to assign a putative function. For this purpose, the 3DM (16) information system was an invaluable *in silico* screen for predicting the experimentally confirmed protease activity of CPR-C4, which can now be applied to the hundreds of other hypothetical proteins sharing extremely high sequence similarity. The remote relationships between such sequences, coupled with structural similarities, are also key to the success of very recent structure prediction tools like AlphaFold2 and RoseTTAFold (41, 42, 43). Both tools were able to generate models of high enough quality for CPR-C4 crystal structure determination by molecular replacement methods (Fig. S9), demonstrating their potential for aiding experimental structure solutions of other hypothetical proteins within the CPR and beyond.

The structure-based multiple sequence alignment methods used in this work result in better quality alignments than classical multiple sequence alignments, and here enabled detailed comparisons of structurally equivalent residues between the homologous CPR-C4 and human VASH1/2 structures, despite the low sequence identity. The conservation of the unusual vasohibin catalytic triad, not only in the CPR-C4 structure, but throughout the CP01A subfamily, suggests that these three residues are also key to the function of CPR-C4 and its homologs throughout the CPR. The structure-based sequence alignments indicate that CPR-C4 is also a cysteine protease, utilizing the same general mechanism and catalytic triad as the VASH1/2 structural homologs. The occurrence of leucine makes this an atypical catalytic triad, and the ability of other amino acids to fulfill its role explains why it is less well conserved than its essential cysteine and histidine counterparts. The distance between the imidazole ring of H93 and carbonyl of L115 in CPR-C4 is larger than in the vasohibins but is still within the acceptable range for H-bonding. Furthermore, these side chain conformations are also dynamic and will change during catalysis.

### Evolutionary relationship between CPR-C4 and human VASH1/2

The discovery of a close structural relationship between CPR-C4 and the human VASH proteins was unexpected, particularly when considering the low sequence identity. While our phylogenetic analysis did not enable identification of a common ancestor for CPR-C4 and VASH1/2, it may suggest a possible, albeit weak, evolutionary link between these proteins. This notion is strongly supported by the overall structural and functional resemblances between the proteins. In particular, the extremely unusual VASH1/2 catalytic triad is conserved in CPR-C4 and its prokaryotic homologs, both in sequence and space. These factors strongly suggest that the proteins are indeed related by divergent evolution, pointing to an important cysteine protease present in species from across the tree of life.

### Potential functional role of CPR-C4 in CPR bacteria

It is well understood that species within the CPR are symbionts, with most having little capability for the *de novo* synthesis of many essential metabolites, including amino acids. However, little is currently known about their interactions with other taxa. A few species are symbiotic with eukaryotes (44), though the abundance and diversity seen in samples with minimal eukaryotic organisms suggests most are likely to be associated with bacteria or archaea. It is probable that the CPR-C4 source organism has a bacterial symbiotic partner, since the vast majority (72.2%) of the 442,373 sequenced genes from the metagenome was bacterial, with only 5.3% archaeal genes, 1.7% eukaryotic genes, and 20.6% from unknown or uncharacterized organisms, alongside 0.2% from viruses.

The CPR-C4 gene appears unrelated to its neighboring genes on the contig (Fig. 1*A*), which makes speculating on a potential function *in vivo* more challenging. The degradation of peptide substrates by CPR-C4 could be necessary to provide essential amino acid metabolites both to the CPR organism and its symbiotic partner. CPR genomes are enriched with pili-related genes compared to other bacteria, and pili-like structures have been observed on the surfaces of CPR cells, which could provide a mechanism for metabolite uptake (45). CPR-C4 could also be involved in the maintenance or regulation of these pili structures.

### A novel protein from metagenomics

This work has identified a novel protease from the CPR and presents the first crystal structure of a protein from a thermophilic CPR bacterium. In the process, a family of novel bacterial proteases featuring an unusual catalytic triad, with hundreds of members across the CPR, has been identified. Roughly 60% of market enzymes worldwide are proteases, with applications throughout several important sectors (46). This work is a significant starting point in the discovery of applications for this unusual cysteine protease, with its potential thermophilic properties making it an interesting candidate for biotechnological applications in future.

A significant hurdle to studying species within the CPR is their inability to be cultured using standard methods. The metagenomic approach, which removes the need for cultivation, has greatly expanded our understanding of microbial life through the discovery of previously inaccessible genomes, such as that containing the CPR-C4 gene (12). With the progression of phylogenetic methods, and an increase in available genome sequences, it is likely that the roles CPR species play in the biosphere, and the extent to which they dominate the bacterial domain, will become clearer in the near future. Many hypothetical proteins with little or no annotation will be recognized to complete metabolic pathways that currently appear broken or missing completely from CPR genomes (2). Isolation and sequencing of additional genomes from a wider variety of sources is necessary to further explore the radiation at the biochemical and structural levels through the emergence of conserved protein families. The fact that roughly 70% of the genes on the CPR-C4 contig have been annotated as hypothetical proteins highlights how poorly understood these genomes are, and the vast inventories of proteins with potentially novel functions that they contain make them enticing targets for further exploration. The recurrence of CPR-C4–type proteases with high sequence conservation throughout the candidate phyla hints at an important function *in vivo* that cannot be confirmed without greater understanding of the lifestyle of CPR organisms.

## Experimental procedures

### Target selection and annotation

The biodiscovery pipeline has previously been outlined in detail (12). Briefly, samples collected from natural environments were processed with consecutive steps of microfiltration, concentration, and DNA extraction in order to isolate genetic material for total metagenomic DNA analysis by next generation sequencing platforms (Illumina MiSeq, HiSeq; Oxford Nanopore). The output from sequencing was assembled and binned for downstream analysis, including quality control and assembly into longer contigs, followed by gene prediction, taxonomic and functional annotation of predicted genes, and binning into metagenome-assembled genomes. Gene products with high innovation potential were selected for expression and characterization, prioritizing those showing extended conservation across genomes but with unknown function.

### Cloning, protein production, and characterization

Cloning of the CPR-C4 gene into the pJOE5751.1 vector (47) and pET28a(+)TEV was conducted by Genscript: the target DNA fragment was inserted *via* BamHI/BsrGI restriction sites to form the pJOE5751.1-*CPRC4* construct and *via* BamH1/Xho1 restriction sites for generation of the pET28a(+)TEV-*CPRC4* construct. Amino acid sequences of the fusion proteins expressed using these constructs are detailed in Fig. S4.

After separate transformations of these constructs into T7 Express Strain competent *E. coli* cells (New England Biolabs), 25 ml precultures were grown overnight in LB at 37 °C (pJOE5751.1-*CPRC4* with 100 μg/ml ampicillin; pET28a(+)TEV-*CPRC4* with 50 μg/ml kanamycin). One liter LB cultures with appropriate antibiotic were inoculated with the preculture and grown (37 °C, 150 rpm) until an OD_600_ of 0.5 was reached, at which point overexpression was induced with the addition of L-rhamnose (0.2%, pJOE5751.1-*CPRC4*) or IPTG (1 mM, pET28a(+)TEV-*CPRC4*) at 25 °C. The cultures were incubated with shaking overnight (25 °C, 150 rpm) and subsequently centrifuged at 1300*g* for 25 min at 4 °C. Cell pellets were resuspended in 20 mM Hepes pH 7.4, 300 mM NaCl, 40 mM imidazole (pJOE5751.1-*CPRC4*), or 20 mM sodium phosphate pH 7.4, 500 mM NaCl, 40 mM imidazole (pET28a(+)TEV-*CPRC4*), with cOmplete Mini EDTA-free Protease Inhibitor Cocktail (Roche), then sonicated and centrifuged at 50,000*g* for 50 min at 4 °C. The supernatant was filtered (0.2 μm) and loaded onto a 1 ml HisTrap HP affinity column (Cytiva) for an imidazole gradient fast protein liquid chromatography separation (40 mM to 500 mM imidazole) using an ÄKTA pure. Protein from the pJOE5751.1-*CPRC4* construct was subsequently incubated overnight at 4 °C with TEV protease for His_6_-tag removal and repurified by imidazole gradient fast protein liquid chromatography separation. CPR-C4 protein molecular weights were verified by electrospray ionization time-of-flight mass spectrometry and SDS-PAGE prior to crystallization and TSA experiments.

Subsequent expression experiments (pJOE5751.1-*CPRC4*) included addition of ZnCl_2_ (100 μM in culture) to LB media for 25 ml precultures and 1 l cultures. Purification by imidazole gradient fast protein liquid chromatography separation was also improved with the use of sodium phosphate buffers (20 mM Na phosphate pH 7.4, 500 mM NaCl, 40 mM to 1 M imidazole gradient).

Size-exclusion analysis was conducted using a HiLoad 16/600 Superdex 75 pg column calibrated using the LMW Calibration Kit (Cytiva) according to instructions provided by the manufacturer. One milliliter of protein sample was injected onto the pre-equilibrated column and was eluted from the column using 20 mM Na phosphate pH 7.4, 500 mM NaCl.

### Thermal shift analysis

TSA was conducted using the Durham Salt and pH Screens (Molecular Dimensions). Protein samples were dialyzed into 10 mM sodium phosphate pH 7.4, 100 mM NaCl. SYPRO orange dye (4 μl, 5000× in DMSO) was added to the protein sample (1 ml, 0.8 mg/ml) to give a protein-plus-dye solution. Ten microliters of Durham Screen condition was added to 10 μl of the protein-plus-dye solution in each well of a standard 96-well PCR plate, which was sealed with thermostable film before centrifugation (2 min, 160*g*). The temperature of the plate was held for 1 min at 1 °C intervals from 24 °C to 96 °C, and fluorescence data for melt temperature, T_m_, experiments were collected with an Applied Biosystems 7500 Fast Real-Time PCR System, with an excitation range of 540 to 550 nm. Data were analyzed with in-house Microsoft Excel scripts and the graphical user interface-based python program NAMI (20) using the emission signal at 567 to 596 nm. Three technical repeats were conducted, with SD used to report experimental variability.

### Crystallization

Purified protein from the pJOE5751.1-*CPRC4* construct, expressed without the addition of ZnCl_2_ to media, was dialyzed into SGC buffer (20 mM Hepes pH 7.4, 300 mM NaCl, 10% v/v glycerol). Initial high-throughput screening was conducted using a Mosquito Crystal protein robot (STP Labtech) and a range of commercially available crystallization screens (Molecular Dimensions). Eighty microliters crystallization reagent was added to reservoirs in MRC 96-well sitting drop plates (Jena Biosciences). Screen conditions and protein samples were combined in two ratios for each screen: 1:1 (100 nl protein: 100 nl crystallization reagent) and 2:1 (200 nl protein: 100 nl crystallization reagent). Plates were observed periodically for crystal formation using a Leica MZ16 Stereomicroscope. Manual crystallization experiments were conducted using the hanging drop vapor diffusion method with 500 μl crystallization reagent in reservoirs and ratios of 1:1 (1 μl: 1 μl) and 2:1 (2 μl: 1 μl) protein solution to crystallization reagent.

Crystal form 1 (dodecahedrons) was obtained at 20 °C using a protein solution of 4 mg/ml in SGC buffer across 25% (24 of 96 conditions) of the Morpheus HT-96 screen and was reproducible in further manual experiments with the Morpheus screen. Crystals predominantly formed in conditions containing a pH 6.5 MES monohydrate buffer, with mixtures of carboxylic acids, amino acids, or salt additives as present in the Morpheus screen. These crystals were fragile and frequently attached to the well surfaces of sitting drop trays. The hanging drop vapor diffusion method was therefore used to make manipulation of the crystals more practicable. Crystals leading to the structural solution of CPR-C4 were grown in 0.09 M NPS, 0.1 M buffer system 1 pH 6.5, 50% v/v precipitant mix 2 from the Morpheus HT-96 screen (Molecular Dimensions).

Crystal form 2 (cubic rods) were grown at 20 °C using 2.4 M sodium malonate dibasic monohydrate pH 7.0 (condition F9 from the JCSG-*plus* HT-96 screen (Molecular Dimensions)). These crystals were more robust and mountable directly from 96-well sitting drop trays.

Crystal form 3 was generated using protein from the pET28a(+)TEV-*CPRC4* construct designed to allow removal of the N-terminal His-tag. Purified protein (after His_6_-tag removal with TEV protease) was dialyzed into phosphate buffer (20 mM sodium phosphate pH 7.4, 500 mM NaCl) for high-throughput crystallization screening with a variety of commercial screens (Molecular Dimensions). Crystals were obtained from a 6 mg/ml protein solution at 20 °C in 0.2 M lithium sulfate, 0.1 M Tris pH 8.5, 15% w/v PEG 4000.

### Data collection, structure solution, and refinement

All crystals were cryoprotected using a 1:1 ratio of glycerol to crystallization solution and flash frozen in liquid nitrogen. Data were collected remotely using beamlines I03 (form 1), I24 (form 2), and I04 (form 3) at the Diamond Light Source. In the case of crystal form 1, native diffraction data were initially processed using autoPROC (24, 25) with STARANISO (24) *via* the ISPyB pipeline, and the autoprocessed data were imported into CCP4i2 (48). Data were phased by multiple wavelength anomalous diffraction at the Zn edge (1.2824 Å, identified with an X-ray absorption edge scan; Fig. S5) and native (0.9763 Å) wavelengths, using the SHELXC/D/E (23) *via* the CRANK2 pipeline (49). Peak data were subsequently reprocessed in XDS (50). Rotational noncrystallographic symmetry was identified between two protein chains in the asymmetric unit; this was exploited through density modification with Parrot (51) applying local noncrystallographic symmetry restraints (52) and a solvent content of 69%, corresponding to two protein molecules in the asymmetric unit. Chain A of the model was built using iterative rounds of manual modifications in *Coot* (53) and REFMAC5 (54) refinement cycles and used to fit chain B by molecular replacement with PHASER (55). The CPR-C4 model was refined using *Coot* (53) and REFMAC5 (54) with jelly-body restraints for initial rounds of refinement and using local noncrystallographic symmetry restraints (52).

Diffraction data for CPR-C4 crystal forms 2 and 3 were processed using xia2 Dials DUI (56) and XDS (50), respectively. The structures were solved by molecular replacement with PHASER (55) using the structure of chain A from crystal form 1 as a homology model. The resulting structures were refined against the density using REFMAC5 (54) with jelly-body restraints for initial rounds of refinement and local noncrystallographic symmetry restraints (52) for crystal form 3, with manual adjustments made in *Coot* (53).

All model building and evaluation was performed with *Coot* (53). The final models were checked using MolProbity (57). Further crystallographic data are summarized in Table 1. Coordinates and structure factors have been deposited in the Protein Data Bank with accession codes 7OB6 (form 1), 7OB7 (form 2), and 7PJO (form 3). Ribbon diagrams were generated using CCP4mg (58). Protein–protein interfaces were analyzed using PISA (26).

### 3DM analysis

A 3DM system (16) for the extended protein superfamily containing CPR-C4 and structural homologs was created using the amino acid sequence and .pdb file of the refined model (form 1 chain A). Two vasohibin structures (VASH1 and VASH2) were identified as sharing structural homology with CPR-C4 through structure superpositions. A structure-based multiple sequence alignment was used to determine conserved ‘core’ regions between these vasohibin structures and CPR-C4, followed by a BLAST (15) search against the three protein sequences resulting in a superfamily multiple sequence alignment containing 2441 sequences in total, including 46 identified during the Virus-X project (1.9%). A synchronized numbering system was used to assign all structurally equivalent residues in the 3DM system the same number (3D number) for direct comparison of corresponding residues between sequences, linking sequence alignment data to the template structures (*H. sapiens* VASH1/2 and CPR-C4 form 1). Many types of protein-related data, including protein-ligand contacts, residue mutations, and protein variants, were extracted from sequences, structures, and the accompanying literature, and collated in the 3DM system. Yasara (59) was used to visualize structural alignment outputs from 3DM.

### Protease activity assays

Protease activity assays were conducted using the commercially available Molecular Probes’ EnzChek Protease Assay Kit (Invitrogen) with green fluorescence from BODIPY-FL casein (33). Purified CPR-C4 was dialyzed into 20 mM sodium phosphate pH 7.4, 500 mM NaCl, 10 mM TCEP. Assays were conducted in a 96-well plate format in Corning nonbinding surface black fluorescence plates using a total reaction volume of 200 μl per well. Hundred microliters of BODIPY-FL casein substrate (10 μg/ml in 20 mM sodium phosphate pH 7.4, 500 mM NaCl) was added to 100 μl protein (concentrations ranging from 0 to 0.05 μM) before centrifugation (2 min, 160*g*). The plates were incubated at 30 °C and fluorescence was read using a Synergy HTX plate reader with a fluorescein filter (ex/em = 485 ± 20 nm/528 ± 20 nm; gain 100). Experiments were carried out with controls for background fluorescence taken in triplicate at each protein concentration without the BODIPY-FL casein substrate in order to account for any intrinsic fluorescent effects of the CPR-C4 protein. Eight technical repeats were conducted at each concentration of CPR-C4. Experimental variability is reported as SD.

### Phylogenetic analysis

Separate BLAST (15) searches were run with the CPR-C4 and *H. sapiens* VASH1/2 sequences using an E value threshold of 0.05. Ninety two representative protein sequences were selected from the results, covering a wide range of taxa and E values, and were aligned using ClustalW (34): 49 from the CPR-C4 search and 43 from VASH1/2 searches. The alignment was used to generate a phylogenetic tree using the Maximum Likelihood method and Jones-Taylor-Thornton matrix-based model in MEGA X (35, 60, 61, 62). Five hundred replicates were used to calculate bootstrap values and used to establish the strength of the consensus tree. National Center for Biotechnology Information accession codes for all sequences can be found in Table S2. Evolutionary analyses were conducted in MEGA X (35).

## Data availability

Metagenomic data and the 3DM systems for CPR-C4 are available upon request. All other data needed to evaluate the conclusions in the paper are present in the article and/or the supporting information. The crystal structures of CPR-C4 have been deposited in the Protein Data Bank under accession codes 7OB6 (form 1), 7OB7 (form 2), and 7PJO (form 3).

## Supporting information

This article contains supporting information.

## Conflict of interest

The authors declare that they have no conflicts of interest with the contents of this article.
