# Supplementary material for: CPR-C4 is a highly conserved novel protease from the Candidate Phyla Radiation with remote structural homology to human vasohibins
Source: J Biol Chem. 2022 Apr 8;298(5):101919. doi: 10.1016/j.jbc.2022.101919 (PMC9108980; doi:10.1016/j.jbc.2022.101919)
Supplement: Supplemental Figures S1–S9, Tables S1 and S2 [file mmc1.pdf]

Supporting information for

CPR-C4 is a highly conserved novel protease from the Candidate Phyla Radiation with remote structural homology to human vasohibins

Katy A. S. Cornish, Joanna Lange, Arnthór Aevarsson, Ehmke Pohl*

*Corresponding author. Email: ehmke.pohl@durham.ac.uk

This PDF file includes:

Figs. S1 to S9
Table S1 and S2

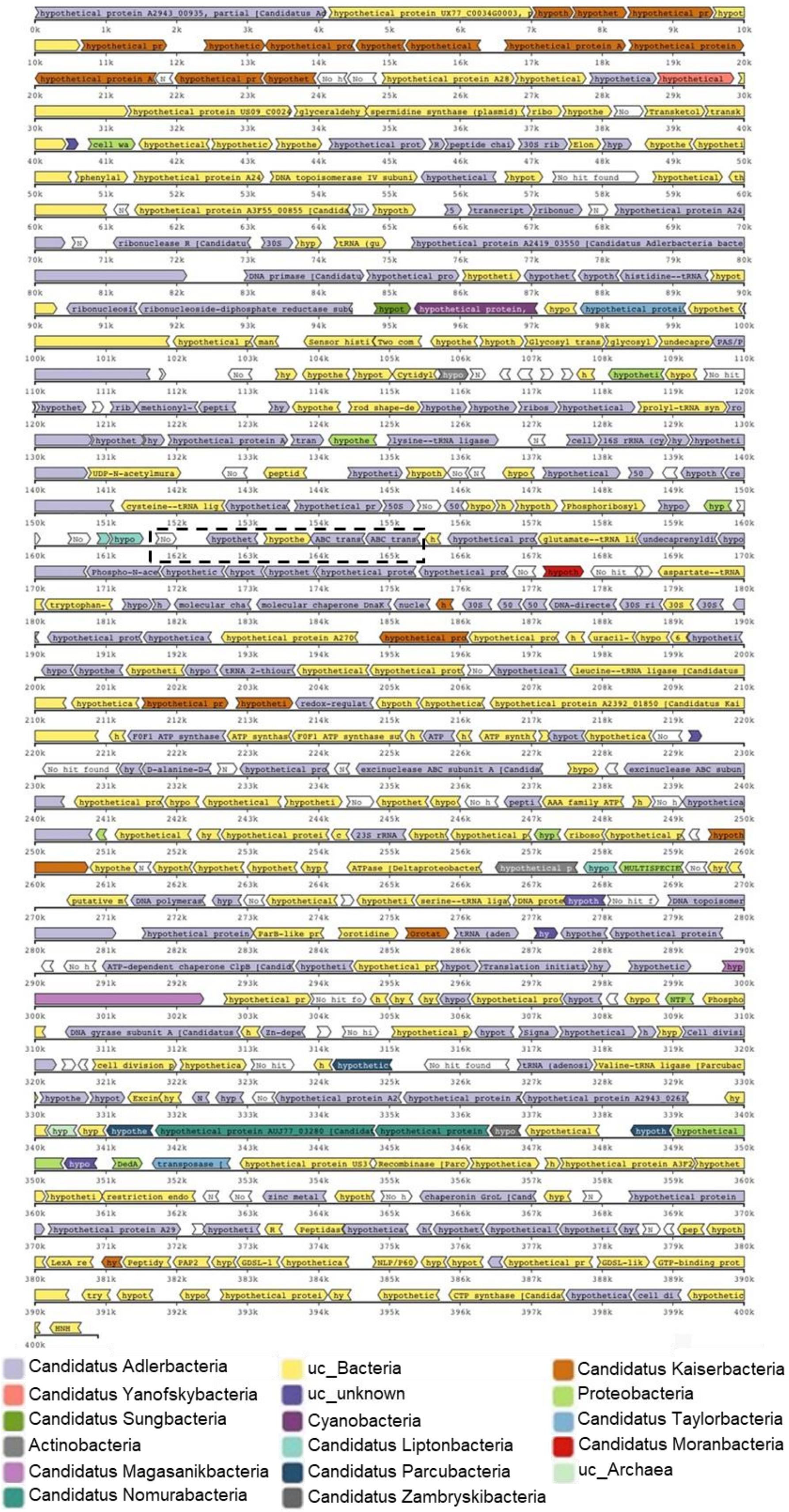
Candidatus Adlerbacteria
Candidatus Yanofskybacteria
Candidatus Sungbacteria
Actinobacteria
Candidatus Magasanikbacteria
Candidatus Nomurabacteria
uc_Bacteria
uc_unknown
Cyanobacteria
Candidatus Liptonbacteria
Candidatus Parcubacteria
Candidatus Zambryskibacteria
Candidatus Kaiserbacteria
Proteobacteria
Candidatus Taylorbacteria
Candidatus Moranbacteria
uc_Archaea

Fig. S1.

Full-length contig containing the CPR-C4 gene, from the contig viewer of the web-based Virus-X project data viewer EMGB2 (*12*). Predicted genes are depicted as arrows following the strand direction for transcription and colour coded by the phylum classification level of their most significant BLAST hit, with the colour key shown underneath; white = unknown phylum. The region containing CPR-C4 and neighbouring genes (Fig. 1A) is indicated with a black dashed box.

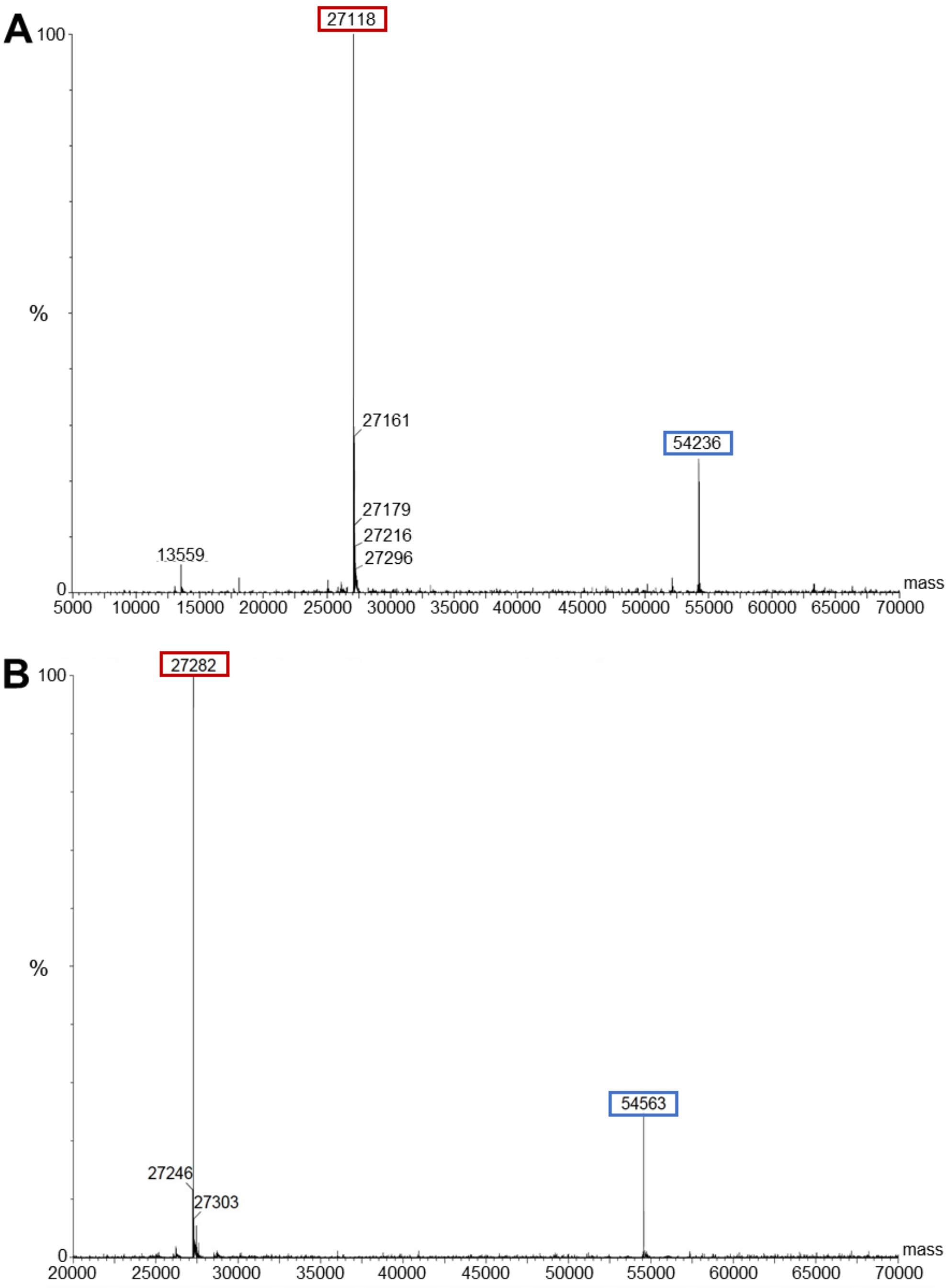

Fig. S2.

(A) Electro-spray ionization time-of-flight (ESI-TOF) mass spectrum of CPR-C4 from the pJOE5751.1-*CPRC4* construct (Fig. S4B). The molecular ion peak at 27116 Da is boxed in red and is within 3 Da of the expected mass (27121 Da), accounting for N-terminal methionine excision. The peak at 54236 Da boxed in blue is the CPR-C4 dimer. **(B)** Mass spectrum of CPR-C4 from the pET28a(+)TEV-*CPRC4* construct after tag cleavage (Fig. S4D) from an ESI-TOF experiment. The molecular ion peak at 27282 Da is boxed in red, and is exactly the expected mass accounting for N-terminal methionine excision. The peak at 54563 Da boxed in blue is the CPR-C4 dimer.

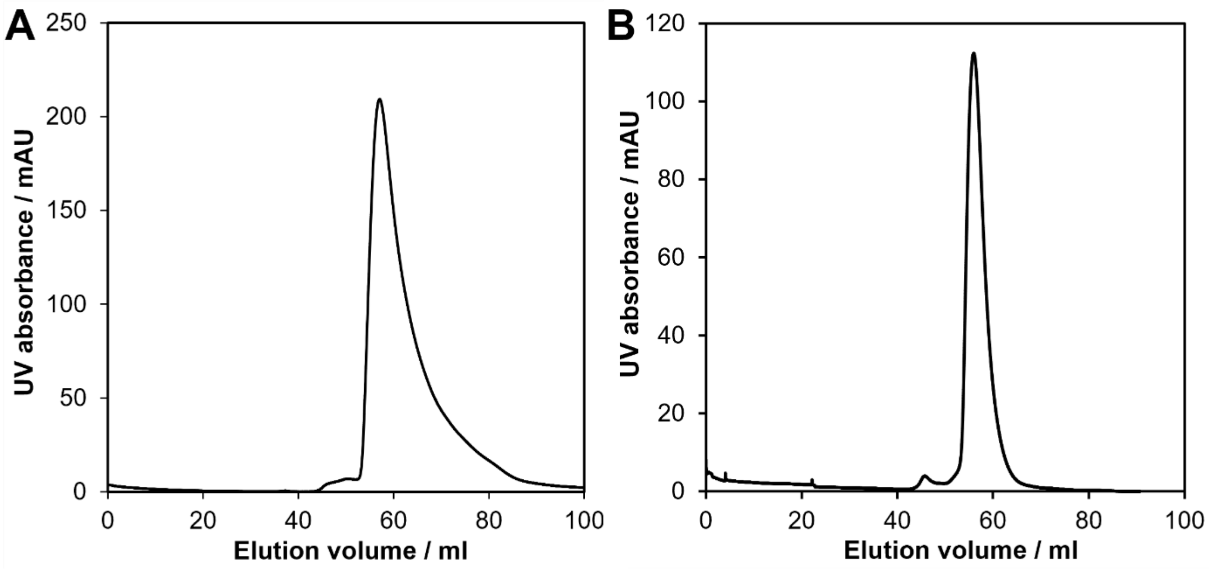

Fig. S3.
(A) Analytical size-exclusion chromatography trace for the CPR-C4 protein from the pJOE5751.1-*CPRC4* construct (Fig. S4B). The peak at 56.8 ml corresponds to a 51.7 kDa moiety, concluded to be dimeric (54.2 kDa) CPR-C4. The peak at 46.4 ml corresponds to a 100.2 kDa moiety, which could be a result of aggregation or a tetramer/dimer of dimers. **(B)** Analytical size-exclusion chromatography trace for the CPR-C4 protein from the pET28a(+)TEV-*CPRC4* construct after tag cleavage (Fig. S4D). The peak at 56.0 ml corresponds to a 54.6 kDa moiety, which is the CPR-C4 dimer (54.6 kDa).

```
A   1  MHYKAQLQKL LTTEEKKILA RLSTPQKIQD FLDTIKNKDL AEGEHTMWSP   50
   51  RAVLKHKHAH CMEGAMLAAL ALAYHGHSPL LMDLQTTDED EDHVVALFKI  100
  101  DGHWGAISKT NHPVLRYRDP IYKSVRELAM SYFHEYFIWW TKKNGGKKTL  150
  151  RAYSNPFDLT RYKPERWVIA TGDLDWLAEA LDDSKHFPIL NKKMQKQLRP  200
  201  ASRIETKAAS LSEWPKRKTN S                                221

B   1  TMITHHHHHH GSMHYKAQLQ KLLTTEEKKI LARLSTPQKI QDFLDTIKNK   50
   51  DLAEGEHTMW SPRAVLKHKH AHCMEGAMLA ALALAYHGHS PLLMDLQTTD  100
  101  EDEDHVVALF KIDGHWGAIS KTNHPVLRYR DPIYKSVREL AMSYFHEYFI  150
  151  WWTKKNGGKK TLRAYSNPFD LTRYKPERWV IATGDLDWLA EALDDSKHFP  200
  201  ILNKKMQKQL RPASRIETKA ASLSEWPKRK TNS                    233

C   1  MGSSHHHHHH SSGENLYFQG HMASMTGGQQ MGRGSMHYKA QLQKLLTTEE   50
   51  KKILARLSTP QKIQDFLDTI KNKDLAEGEH TMWSPRAVLK HKHAHCMEGA  100
  101  MLAALALAYH GHSPLLMDLQ TTDEDEDHVV ALFKIDGHWG AISKTNHPVL  150
  151  RYRDPIYKSV RELAMSYFHE YFIWWTKKNG GKKTLRAYSN PFDLTRYKPE  200
  201  RWVIATGDLD WLAEALDDSK HFPILNKKMQ KQLRPASRIE TKAASLSEWP  250
  251  KRKTNS                                                 256

D   1  GHMASMTGGQ QMGRGSMHYK AQLQKLLTTE EKKILARLST PQKIQDFLDT   50
   51  IKNKDLAEGE HTMWSPRAVL KHKHAHCMEG AMLAALALAY HGHSPLLMDL  100
  101  QTTDEDEDHV VALFKIDGHW GAISKTNHPV LRYRDPIYKS VRELAMSYFH  150
  151  EYFIWWTKKN GGKKTLRAYS NPFDLTRYKP ERWVIATGDL DWLAEALDDS  200
  201  KHFPILNKKM QKQLRPASRI ETKAASLSEW PKRKTNS                237
```

Fig. S4.

Amino acid sequences of the CPR-C4 fusion proteins from this study. Non-native tag regions are shown in red, with His_6-tags in bold. Catalytic triad residues are highlighted in yellow; aspartate residues from the Zn^{2+} binding site (crystal form 1 chain A) are highlighted in blue. **(A)** Native CPR-C4 sequence. **(B)** CPR-C4 fusion protein from the pJOE5751.1-*CPRC4* construct. **(C)** Full-length CPR-C4 fusion protein from the pET28a(+)TEV-*CPRC4* construct. The TEV protease recognition sequence is underlined, with the cleavage site indicated with a black triangle. **(D)** CPR-C4 fusion protein from the pET28a(+)TEV-*CPRC4* construct after cleavage with TEV protease.

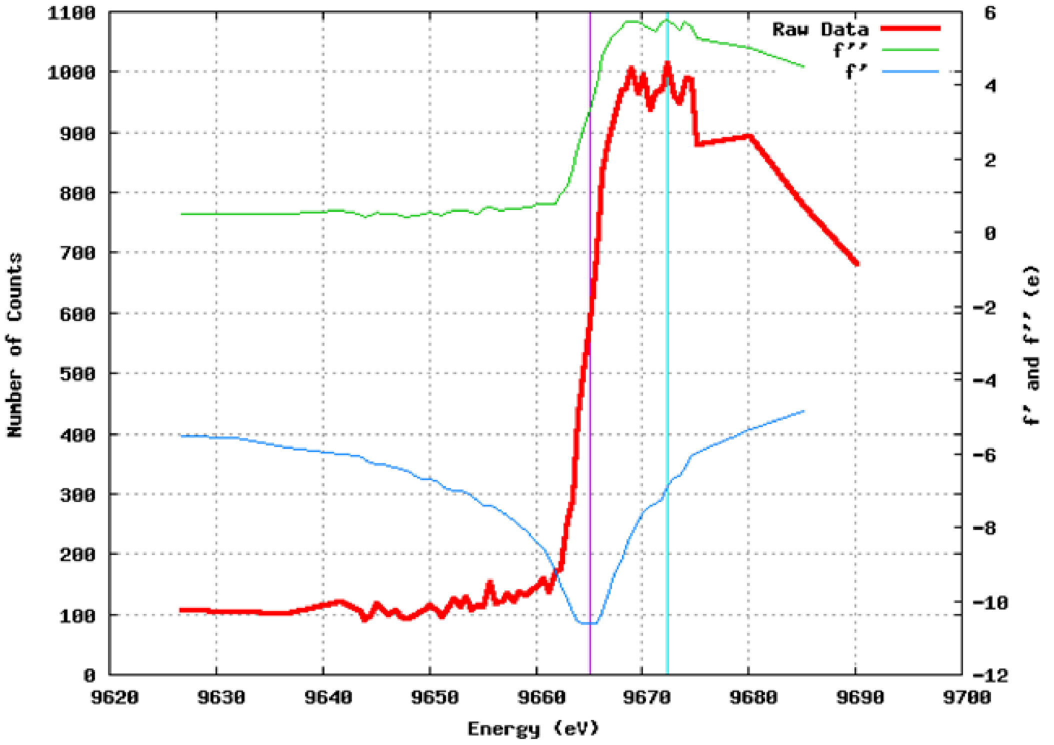

Fig. S5.

UV fluorescence scan at the Zn edge of CPR-C4 form 1 crystals, conducted at DLS I03, in order to select wavelengths for diffraction data collection to facilitate multiple wavelength anomalous diffraction phasing. The raw data trace is shown in red; the f'' curve is shown in green; the f' curve is shown in blue. The peak energy is indicated with a cyan vertical line; the inflection energy is indicated with a pink vertical line.

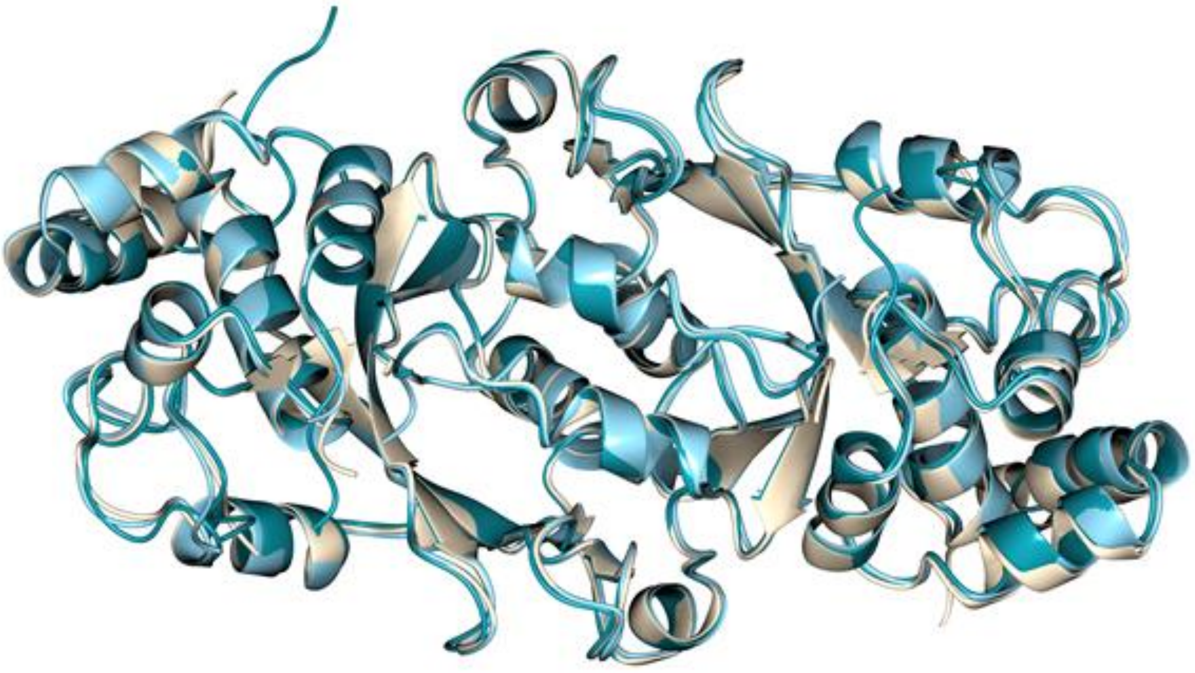

Fig. S6.

Ribbon diagram of the least-squares superposition of CPR-C4 dimers from the three crystal forms: form 1 shown in teal, form 2 in light blue, and form 3 in white; Zn^{2+} ions found in form 1 chain A only have been omitted for clarity.

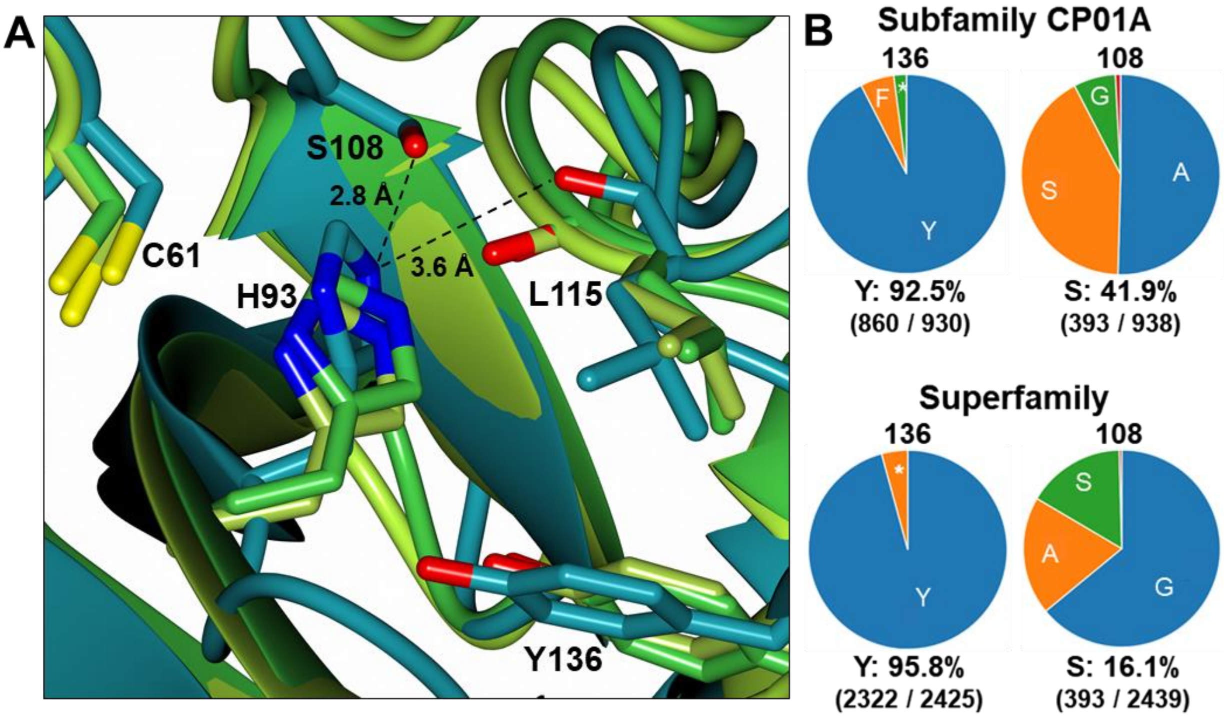

Fig. S7.

(A) Catalytic triad residues in CPR-C4 (form 1 chain A, teal) aligned with equivalent 3D residues from human VASH1 (6J8F chain B, green) and VASH2 (6J4P chain A, yellow green) through least-squares superposition. The positions of the conserved tyrosine residue (Y136) and proximal serine residue (S108) are indicated. The main chain is shown with ribbon representation; specified side chains and the backbone carbonyl group of L115 are shown with cylinder representation, O atoms in red, N atoms in blue, S atoms in yellow; interatomic distances in Å are indicated with dashed lines. **(B)** Conservation of the tyrosine and serine residues proximal to the CPR-C4 catalytic triad across the CP01A subfamily and the extended superfamily comprising the CP01A, 6J4PA and 6J8FB subfamilies; * indicates all other residue identities.

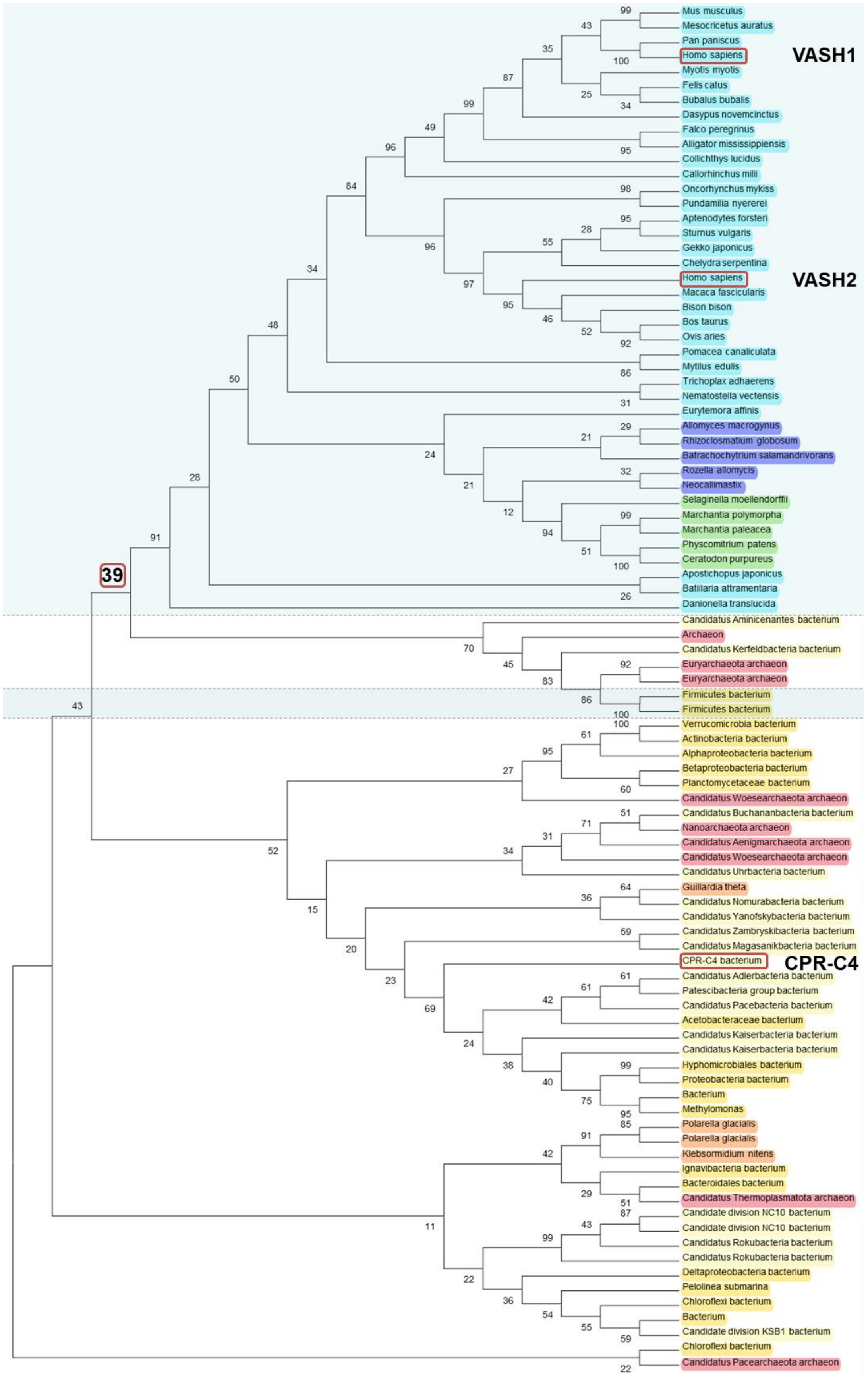
VASH1
VASH2
CPR-C4
39
Mus musculus
Mesocricetus auratus
Pan paniscus
Homo sapiens
Myotis myotis
Felis catus
Bubalus bubalis
Dasypus novemcinctus
Falco peregrinus
Alligator mississippiensis
Collichthys lucidus
Callorhinchus milii
Oncorhynchus mykiss
Pundamilia nyererei
Aptenodytes forsteri
Sturnus vulgaris
Gekko japonicus
Chelydra serpentina
Homo sapiens
Macaca fascicularis
Bison bison
Bos taurus
Ovis aries
Pomacea canaliculata
Mytilus edulis
Trichoplax adhaerens
Nematostella vectensis
Eurytemora affinis
Allomyces macrogynus
Rhizoclosmatium globosum
Batrachochytrium salamandrivorans
Rozella allomycis
Neocallimastix
Selaginella moellendorffii
Marchantia polymorpha
Marchantia paleacea
Physcomitrium patens
Ceratodon purpureus
Apostichopus japonicus
Batillaria attramentaria
Danionella translucida
Candidatus Aminicenantes bacterium
Archaeon
Candidatus Kerfeldbacteria bacterium
Euryarchaeota archaeon
Euryarchaeota archaeon
Firmicutes bacterium
Firmicutes bacterium
Verrucomicrobia bacterium
Actinobacteria bacterium
Alphaproteobacteria bacterium
Betaproteobacteria bacterium
Planctomycetaceae bacterium
Candidatus Woesearchaeota archaeon
Candidatus Buchananbacteria bacterium
Nanoarchaeota archaeon
Candidatus Aenigmarchaeota archaeon
Candidatus Woesearchaeota archaeon
Candidatus Uhrbacteria bacterium
Guillardia theta
Candidatus Nomurabacteria bacterium
Candidatus Yanofskybacteria bacterium
Candidatus Zambryskibacteria bacterium
Candidatus Magasanikbacteria bacterium
CPR-C4 bacterium
Candidatus Adlerbacteria bacterium
Patescibacteria group bacterium
Candidatus Pacebacteria bacterium
Acetobacteraceae bacterium
Candidatus Kaiserbacteria bacterium
Candidatus Kaiserbacteria bacterium
Hyphomicrobiales bacterium
Proteobacteria bacterium
Bacterium
Methylomonas
Polarella glacialis
Polarella glacialis
Klebsormidium nitens
Ignavibacteria bacterium
Bacteroidales bacterium
Candidatus Thermoplasmatota archaeon
Candidate division NC10 bacterium
Candidate division NC10 bacterium
Candidatus Rokubacteria bacterium
Candidatus Rokubacteria bacterium
Deltaproteobacteria bacterium
Pelolinea submarina
Chloroflexi bacterium
Bacterium
Candidate division KSB1 bacterium
Chloroflexi bacterium
Candidatus Pacearchaeota archaeon

Fig. S8.

Phylogenetic analysis of CPR-C4 and human VASH1/2 proteins. Bootstrap consensus tree generated in MEGA X using protein sequences related to CPR-C4 and human VASH1/2 after alignment in ClustalW. Sequences from BLAST searches of the human VASH1/2 proteins are indicated with a pale blue background to distinguish from sequences resulting from BLAST of CPR-C4. Branches are labelled with the species name of the host organism where known, and color coded according to taxonomic classification: Animalia are colored blue, Plantae in green, Fungi in dark blue, Algae in orange, Archaea in red, Bacteria in yellow, with bacteria from the CPR in pale yellow. The locations of human VASH1/2 and CPR-C4 within the tree are indicated with labelled red boxes. The evolutionary history was inferred using the Maximum Likelihood method and Jones-Taylor-Thornton matrix-based model using MEGA X. Bootstrap values from 500 replicates are shown next to each branch and indicate the percentage of replicate trees in which the associated taxa clustered together. The bootstrap value of 39% at the node linking CPR-C4-related sequences to human VASH1/2-related sequences is highlighted in grey. NCBI accession numbers for all sequences used can be found in Table S2.

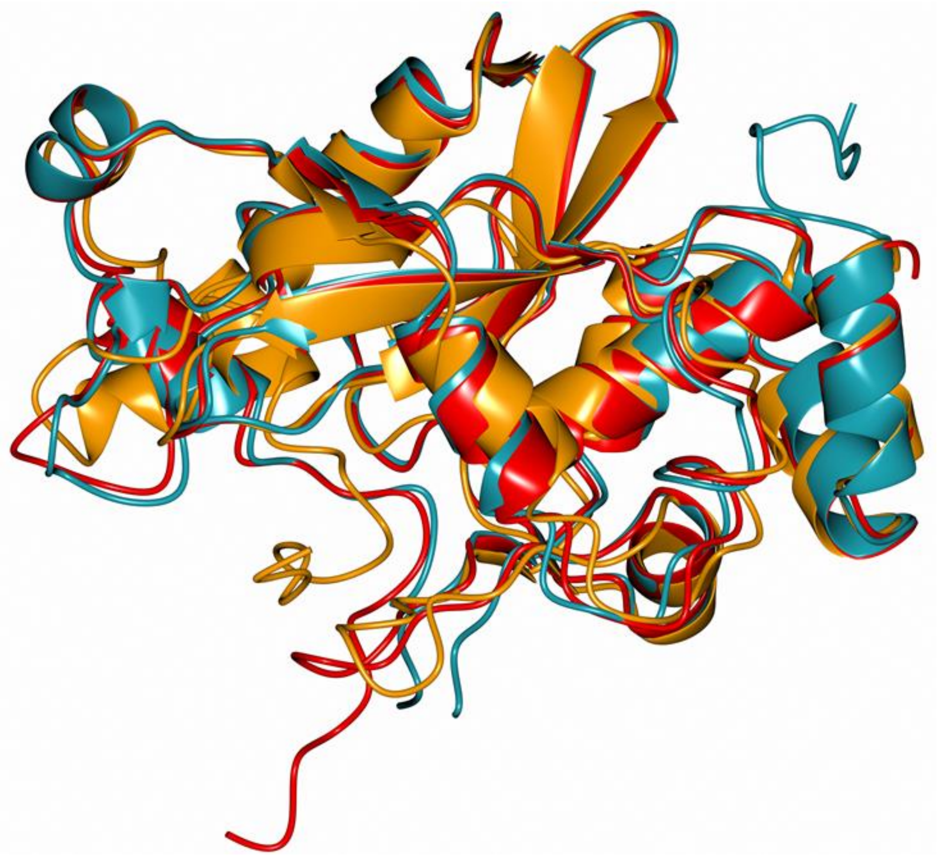

Fig. S9.
Ribbon diagram showing the alignment of the experimentally determined CPR-C4 structure (form 1 chain A, teal) with models generated using AlphaFold2 (red, RMSD 0.84 Å) and RoseTTAFold (orange, RMSD 1.95 Å) through least-squares superposition.

Table S1.

Matrix of RMSD values in Å between the CPR-C4 chains from the three crystal forms; numbers of residues aligned are shown in parentheses.

	**7OB6 chain A**	**7OB6 chain B**	**7OB7**	**7PJO chain A**	**7PJO chain B**
**7OB6 chain A**		**0.52 (213)**	**0.37 (209)**	**0.42 (211)**	**0.64 (213)**
**7OB6 chain B**	**0.52 (213)**		**0.27 (209)**	**0.22 (211)**	**0.25 (211)**
**7OB7**	**0.37 (209)**	**0.27 (209)**		**0.31 (209)**	**0.31 (209)**
**7PJO chain A**	**0.42 (211)**	**0.22 (211)**	**0.31 (209)**		**0.27 (215)**
**7PJO chain B**	**0.64 (213)**	**0.25 (211)**	**0.31 (209)**	**0.27 (215)**	

Table S2.

NCBI accession codes for the protein sequences used in phylogenetic tree construction (Fig. S8). Taxonomic classification is at the species level where known.

#	Accession code	Taxonomy	#	Accession code	Taxonomy
1	NP 796328.2	*Mus musculus*	47	HEK51315.1	*Firmicutes bacterium*
2	XP 005084063.1	*Mesocricetus auratus*	48	HEQ02367.1	*Firmicutes bacterium*
3	XP 003808906.1	*Pan paniscus*	49	GDY17089.1	*Verrucomicrobia bacterium*
4	sp|Q7L8A9	*Homo sapiens*	50	NCZ71312.1	*Actinobacteria bacterium*
5	XP 036169856.1	*Myotis myotis*	51	MBM3582227.1	*Alphaproteobacteria bacterium*
6	XP 003987908.1	*Felis catus*	52	TMH00807.1	*Betaproteobacteria bacterium*
7	XP 006052929.1	*Bubalus bubalis*	53	MBT6460655.1	*Planctomycetaceae bacterium*
8	XP 004453621.1	*Dasypus novemcinctus*	54	HIH23410.1	*Candidatus Woesearchaeota archaeon*
9	XP 013159882.1	*Falco peregrinus*	55	OGY49875.1	*Candidatus Buchananbacteria bacterium*
10	XP 006274820.2	*Alligator mississippiensis*	56	MBU0958765.1	*Nanoarchaeota archaeon*
11	TKS86491.1	*Collichthys lucidus*	57	MBM3303958.1	*Candidatus Aenigmarchaeota archaeon*
12	XP 007910324.1	*Callorhinchus milii*	58	MBI4152238.1	*Candidatus Woesearchaeota archaeon*
13	XP 021467687.1	*Oncorhynchus mykiss*	59	KAA0206149.1	*Candidatus Uhrbacteria bacterium*
14	XP 005753174.2	*Pundamilia nyererei*	60	XP 005825204.1	*Guillardia theta*
15	XP 009277863.1	*Aptenodytes forsteri*	61	HCY17607.1	*Candidatus Nomurabacteria bacterium*
16	XP 014732398.1	*Sturnus vulgaris*	62	OGN19854.1	*Candidatus Yanofskybacteria bacterium*
17	XP 015283097.1	*Gekko japonicus*	63	PIP56008.1	*Candidatus Zambryskibacteria bacterium*
18	KAG6928815.1	*Chelydra serpentina*	64	MBI2038209.1	*Candidatus Magasanikbacteria bacterium*
19	sp|Q86V25	*Homo sapiens*	65	(CPR-C4)	*CPR-C4 bacterium*
20	XP 005540865.1	*Macaca fascicularis*	66	KKW35909.1	*Candidatus Adlerbacteria bacterium*
21	XP 010851343.1	*Bison bison*	67	MBI2004736.1	*Patescibacteria group bacterium*
22	NP 001073079.1	*Bos taurus*	68	MBP7770728.1	*Candidatus Pacebacteria bacterium*
23	XP 027812095.1	*Ovis aries*	69	MBC7836912.1	*Acetobacteraceae bacterium*
24	PVD25902.1	*Pomacea canaliculata*	70	MBI4093848.1	*Candidatus Kaiserbacteria bacterium*
25	CAG2227822.1	*Mytilus edulis*	71	OGG53456.1	*Candidatus Kaiserbacteria bacterium*
26	XP 002109112.1	*Trichoplax adhaerens*	72	MBI3702436.1	*Hyphomicrobiales bacterium*
27	XP 032237246.1	*Nematostella vectensis*	73	MBS0533742.1	*Proteobacteria bacterium*
28	XP 023340823.1	*Eurytemora affinis*	74	MBL8158630.1	*Bacterium*
29	KNE57617.1	*Allomyces macrogynus*	75	MBS4050093.1	*Methylomonas*
30	ORY40458.1	*Rhizoclosmatium globosum*	76	CAE8679133.1	*Polarella glacialis*
31	OON07946.1	*Batrachochytrium salamandrivorans*	77	CAE8638246.1	*Polarella glacialis*
32	EPZ36457.1	*Rozella allomycis*	78	GAQ84263.1	*Klebsormidium nitens*
33	KAG4090764.1	*Neocallimastix*	79	OGU65384.1	*Ignavibacteria bacterium*
34	XP 024530086.1	*Selaginella moellendorffii*	80	HBH84536.1	*Bacteroidales bacterium*
35	OAE33039.1	*Marchantia polymorpha*	81	MBN1539844.1	*Candidatus Thermoplasmatota archaeon*
36	KAG6549164.1	*Marchantia paleacea*	82	MBI1999858.1	*Candidate division NC10 bacterium*
37	XP 024370159.1	*Physcomitrium patens*	83	MBI2000069.1	*Candidate division NC10 bacterium*
38	KAG0629661.1	*Ceratodon purpureus*	84	PYN57126.1	*Candidatus Rokubacteria bacterium*
39	PIK60116.1	*Apostichopus japonicus*	85	PYM30278.1	*Candidatus Rokubacteria bacterium*
40	KAG5702141.1	*Batillaria attramentaria*	86	MBN2498250.1	*Deltaproteobacteria bacterium*
41	TRY54892.1	*Danionella translucida*	87	WP 116225417.1	*Pelolinea submarina*
42	MBP1660180.1	*Candidatus Aminicenantes bacterium*	88	MBM3152234.1	*Chloroflexi bacterium*
43	AJF62464.1	*Archaeon*	89	MBK7703915.1	*Bacterium*
44	MBI4414812.1	*Candidatus Kerfeldbacteria bacterium*	90	MBN1995914.1	*Candidate division KSB1 bacterium*
45	OGS51393.1	*Euryarchaeota archaeon*	91	MBC7257623.1	*Chloroflexi bacterium*
46	TLZ64862.1	*Euryarchaeota archaeon*	92	MBI2044722.1	*Candidatus Pacearchaeota archaeon*
